# Cannabidiol for Pain Treatment: Focus on Pharmacology and Mechanism of Action

**DOI:** 10.3390/ijms21228870

**Published:** 2020-11-23

**Authors:** Jakub Mlost, Marta Bryk, Katarzyna Starowicz

**Affiliations:** Department of Neurochemistry, Maj Institute of Pharmacology, Polish Academy of Sciences, 31-343 Krakow, Poland; mlost@if-pan.krakow.pl (J.M.); bryk@if-pan.krakow.pl (M.B.)

**Keywords:** CBD, network pharmacology, pain, neuropathic, osteoarthritis

## Abstract

Cannabis has a long history of medical use. Although there are many cannabinoids present in cannabis, Δ9tetrahydrocannabinol (Δ9-THC) and cannabidiol (CBD) are the two components found in the highest concentrations. CBD itself does not produce typical behavioral cannabimimetic effects and was thought not to be responsible for psychotropic effects of cannabis. Numerous anecdotal findings testify to the therapeutic effects of CBD, which in some cases were further supported by research findings. However, data regarding CBD’s mechanism of action and therapeutic potential are abundant and omnifarious. Therefore, we review the basic research regarding molecular mechanism of CBD’s action with particular focus on its analgesic potential. Moreover, this article describes the detailed analgesic and anti-inflammatory effects of CBD in various models, including neuropathic pain, inflammatory pain, osteoarthritis and others. The dose and route of the administration-dependent effect of CBD, on the reduction in pain, hyperalgesia or allodynia, as well as the production of pro and anti-inflammatory cytokines, were described depending on the disease model. The clinical applications of CBD-containing drugs are also mentioned. The data presented herein unravel what is known about CBD’s pharmacodynamics and analgesic effects to provide the reader with current state-of-art knowledge regarding CBD’s action and future perspectives for research.

## 1. Introduction

*Cannabis sativa* L. has a long history of medical use with some of the earliest records found in Egyptian medical papyrus dating circa 1550 BC [1]. Although there are many cannabinoids present in *Cannabis* sp., Δ^9^tetrahydrocannabinol (Δ9-THC) and cannabidiol (CBD) are the two components found at the highest concentrations (for review of complex interactions between phytocannabinoids [2]). Δ9-THC is the primary psychoactive ingredient of cannabis with an established partial agonist activity on both of the canonical cannabinoid receptors, CB1 and CB2. Therefore, Δ9-THC has been a natural subject of thousands of studies concerning pharmacology, toxicity and the therapeutic potential of cannabinoids in the intervening years. This research led to the discovery of the endocannabinoid system, which consists of G-protein coupled (GPCR) cannabinoid receptors and their endogenous ligands, endocannabinoids, anandamide (AEA) and 2-arachidonoylglycerol (2AG). Endocannabinoids are lipid compounds that are metabolized in multiple enzymatic pathways involving specific enzymes, such as fatty acid amide hydrolase (FAAH), monoacylglycerol lipase or alternative pathways involving non-specific enzymes, often interlinked with inflammatory mediators, such as cyclooxygenases or lipoxygenases (for review of endocannabinoid system metabolism pathways [3]). This interaction between metabolic pathways occurs due to the structural similarity between prostaglandins and endocannabinoids. However, the primary effects of endocannabinoids depend upon GPCR activation and the type of α subunit coupled with the receptor. CB1 and CB2 are primary couple with the Gαi/o subunit, which inhibits adenylate cyclase activity and therefore lowers intracellular Ca^2+^ concentration. Despite the specificity of the endocannabinoid mechanism of action, complex metabolic interactions between endocannabinoid system and inflammatory mediators need to be taken into consideration as well when studying the effects of various cannabinoid-based drugs.

Although isolated earlier [4] than Δ9-THC, CBD has remained a more elusive and poorly studied substance, because CBD itself does not produce typical behavioral cannabimimetic effects, and was thought not to be responsible for psychotropic effects of cannabis [5]. The complicated legal status of CBD, throughout the world, further restricted the research and professional knowledge about the therapeutic potential of this compound. In spite of the limitations, numerous anecdotal findings testify to the therapeutic effects of CBD, including anticonvulsant, antipsychotic, anxiolytic, neuroprotective and sleep-promoting effects, which are further supported by research [6]. Initial clinical evidence suggests that CBD possesses a desirable safety profile [7], while numerous preclinical findings present anti-inflammatory effects of CBD [8,9,10,11,12]. However, the pharmacodynamics of CBD have been difficult to elucidate. Initial reports demonstrated that CBD competes poorly with cannabinoid ligands at the orthosteric site of cannabinoid receptors [13], leading to the conclusion that any action of CBD occurs independent of cannabinoid receptors. Further studies revealed this conclusion as partially true. Indeed, CBD directly interacts with various receptors, enzymes and ion channels; however, it was also shown to directly and indirectly interact with the endocannabinoid system [14].

Taken together, these initial findings imply that CBD represents a promising phytocannabinoid-based treatment option. However, data regarding its mechanism of action and therapeutic potential are abundant and omnifarious. Therefore, we review the basic research regarding the molecular mechanisms of CBD’s actions with particular focus on its analgesic potential. The data presented herein unravel what is known about CBD’s pharmacodynamics and analgesic effects to provide readers with current state-of-art knowledge regarding CBD’s action and future perspectives for research. A summary of the data discussed herein will be shared as Supplementary Materials.

## 2. Mechanism of Action

### 2.1. GPCRs

As mentioned above, CBD’s binding to the orthosteric site of cannabinoid receptors is weak, with most studies reporting a Ki in the micromolar range [14,15,16,17,18] with one exception presenting Ki = 34 nM and partial agonism of CBD of the human CB2 receptors in HEK293A cells [14]. Surprisingly, much lower doses of CBD, in the nanomolar range, were able to antagonize the effects elicited by orthosteric agonists of CB1 and CB2, CP55940 and *R*-(+)-WIN55212 [17]. Recent developments in pharmacological studies explained this phenomenon through negative allosteric modulation of the cannabinoid receptors [14,18]. Moreover, it should be noted that CBD coadministration in vitro skews the signaling bias of various cannabinoid ligands [19,20].

The current state of knowledge has exposed CBD’s mechanism action as distinct from the endocannabinoid system. Studies often present CBD’s effects to be mediated by the serotoninergic 5HT1a receptor (5HT1a), which, similar to cannabinoid receptors, is coupled to the Gi protein. Although CBD’s binding to the orthosteric site of 5HT1a is relatively weak (Ki = 16 mM [21]), 100 nM CBD produced an upward shift in the log concentration response curve of the 5HT1a agonist 8-OH-DPAT that resulted in a statistically significant increase in the Emax [22], suggesting positive allosteric modulation of 5HT1a.

CBD also interacts with various orphan GPCRs (GPR). CBD is antagonist for GPR55 (EC50 = 445 nM) [23] coupled with G_13_ alpha protein, regulating actin cytoskeletal remodeling in cells during movement and migration [24]. GPR55 has also been shown by other methods to use G_q_, G_12_, or G_13_ for signal transduction, resulting in downstream activation of RhoA and PLC [24,25,26]. This signaling mode is associated with temporal changes in cytoplasmic calcium, membrane-bound diacylglycerol, and plasma membrane topology. GPR55 is widely expressed in the brain, but its physiological function is still unclear. CBD is also an inverse agonist for GPR3, GPR6 and GPR12, with EC50 values of 1220 nM, 180 nM and 10,000 nM, respectively [27,28]. GPR3, GPR6 and GPR12 are primarily responsible for neurite outgrowth, cell survival and proliferation but have also been implicated in neuropathic pain development [29].

**Figure 1:**
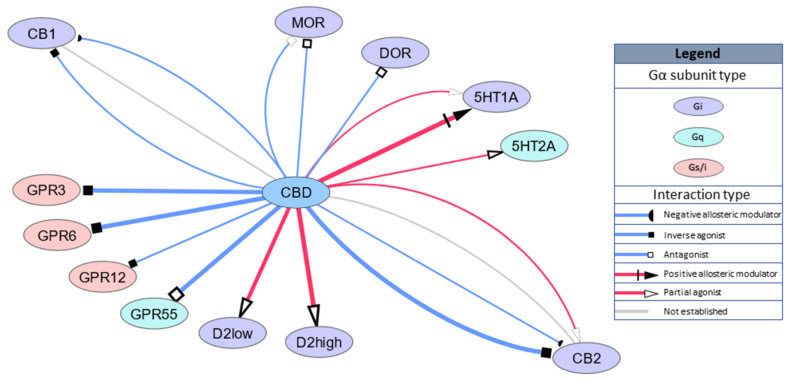
Schematic representation of cannabidiol (CBD) GPCR targets. Width of the edges (lines) represent relative affinity or EC/IC50 for the target (range 11–1000 nM).

Studies have also revealed that CBD binds to other Gi-coupled receptors, namely, opioid receptors, μ-opioid receptor (MOR) and δ-opioid receptor (DOR) with Ki = 7000 nM and Ki = 10,000 nM, respectively, or Ki = 11 nM for D2 receptors with functional high-affinity for dopamine. A recent computational study revealed that the dopamine receptor D3 is a novel predicted target for CBD action [30]. GPCR targets are schematically summarized in Figure 1.

### 2.2. Ionotropic Receptors

**Figure 2:**
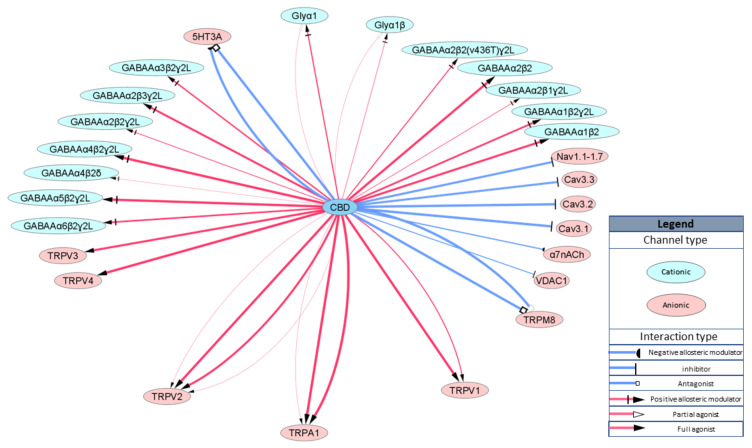
Schematic representation of CBD ionotropic targets. Width of the edges (lines) represents relative EC/IC50 ranging between 60 and 20,000 nM.

High activity for ionotropic receptors is a significant contributor to the physiological effects of CBD. Transient receptor potential (TRP) channels are a group of cationic ion channels primarily localized on the plasma membrane of numerous animal cell types. CBD activates TRPA1, TRPV1 and TRPV4 at low nanomolar concentrations [31,32,33] and TRPV2 at high micromolar concentrations [31,34,35]. Moreover, CBD is an antagonist for TRPM8 with an IC50 approximately 80 nM [31,32] and negative allosteric modulator for serotonin receptor 5HT3a (IC50 = 600 nM, [36]) and α7 nicotinic acetylcholine receptor (IC50 = 11,300 nM, [37]), which are also selective for positively charged ions. On the other hand, CBD is also a positive allosteric modulator in micromolar concentration ranges for anionic ligand-gated ion channels, such as GABA_A_ [38] and glycine receptors (GlyRs) [39]. CBD also inhibits cationic currents through voltage gated calcium channels Cav3.1 and Cav3.2 with IC50 approximately 800 nM [40] and voltage-gated sodium channels and Cav3.3 with IC50 approximately 3000 nM [40,41]. CBD has also been shown to directly interact with voltage-dependent anion channel 1 (VDAC1), decreasing its conductance [42]. Ionotropic targets are schematically summarized in Figure 2.

### 2.3. Transporters

**Figure 3:**
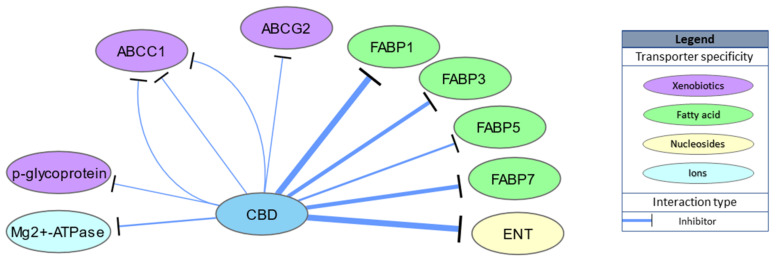
Schematic representation of CBD transporter targets. Width of the edges (lines) represents relative EC/IC50 ranging between 100 and 10,000 nM.

CBD is also able to bind intracellular transporters of endocannabinoids, including fatty acid binding proteins 1, 3, 5 and 7 (Ki = 167, 1690, 1880 and 1520 nM, respectively), indirectly enhancing endocannabinoid actions through inhibition of anandamide uptake [43,44]. Indeed, CBD administration increases AEA levels in rat brains in an N-acyl phosphatidylethanolamine phospholipase D dependent manner [45] and in human serum [46]. Moreover, low nanomolar concentrations of CBD (IC50 = 124 nM) inhibit equilibrative nucleoside transporter (ENT) and adenosine uptake, which underlies both the anti-inflammatory and possibly sleep promoting effects of CBD [47,48]. CBD activity for other transporter proteins, such as multidrug resistance proteins (multidrug resistance-associated protein 1 (ABCC1), ATP-binding cassette super-family G member 2 (ABCG2) or P-glycoprotein) or Mg2+-ATPase, has also been studied; however, IC50 values above 3000 nM suggest these to be physiologically irrelevant. Transporter targets are schematically summarized in Figure 3.

### 2.4. Enzymes

**Figure 4:**
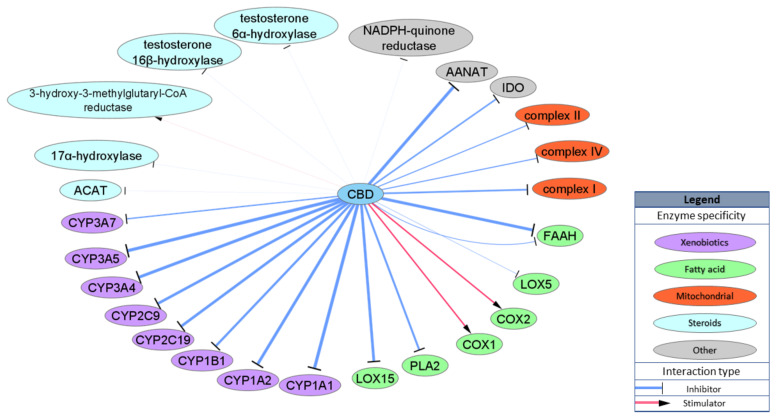
Schematic representation of CBD enzyme targets. Width of the edges (lines) represents relative EC/IC50 ranging between 77 and 30,000 nM.

Another important group of CBD targets is enzymes. The cytochrome P450 superfamily (CYPs) of enzymes is of particular interest as the interaction between CBD and CYPs may influence clearance of various drugs, including very commonly used, non-steroidal anti-inflammatory drugs. It has been shown that CBD binds and inhibits activity of various CYPs, including CYP1B1, CYP2C19, CYP2C9, CYP3A4 and CYPC3A7, in physiologically relevant concentrations, with prominent IC50 values for CYP1A1 and CYP3A5 (IC50 = 77 and 195 nM, respectively). Due to the structural properties of CBD, it may also interact with various enzymes involved in lipid metabolism, most of which are involved in AEA metabolism. CBD inhibits rat FAAH activity with IC50 = 1520 nM [31], but the IC50 for the human isoform of FAAH is over 25,000 nM [33], which excludes FAAH as a possible target for CBD activity in humans. On the other hand, CBD inhibits alternative and nonspecific enzymes lipooxygenases (LOXs) involved in AEA degradation and production of inflammatory factors from arachidonic acid: LOX15 with IC50 = 2560 nM and LOX5 with IC50 = 73,730 nM [49]. Other studies have presented CBD as a stimulator of ovine COX1 and human recombinant COX2 with an IC50 approximately 10,000 nM [50] or as an inhibitor of phospholipase A2 (PLA2) from *Naja naja* venom with an IC50 = 6400 nM [51]. CBD has also been shown to inhibit mitochondrial complex I, II and IV but with very low potency (IC50 = 8200–19,100 nM) [52]. Moreover, CBD inhibits enzyme involved in the conversion of serotonin to melatonin, aralkylamine N-acetyltransferase (AANAT) with IC50 = 1000 nM [53], and an enzyme involved in tryptophan catabolism, indoleamine-pyrrole 2,3-dioxygenase (IDO) with IC50 = 8900 nM [54]. Few studies have examined CBD’s interaction with enzymes involved in steroid metabolism such as acyl-CoA cholesterin acyltransferase (ACAT) or testosterone hydroxylase; however, either IC50 values were not specified or the concentration that was utilized extended beyond physiologically relevant values [55,56]. Enzymatic targets are schematically summarized in Figure 4.

### 2.5. Nuclear Factors

One of the more important targets of CBD is the nuclear receptor peroxisome proliferator-activated receptor gamma (PPARγ), especially in the context of inflammation. First, CBD has been shown to have low potency as a full agonist (EC50 = 20,100 nM) [57]; albeit a later study by Hedge et al., established an EC50 approximately 100 nM [58]. Moreover, studies by Juknat et al. revealed that nuclear factor erythroid-derived 2-like 2 (Nrf2) was responsible for CBD-mediated modulation of inflammatory gene expression patterns in activated microglia cells [59].

### 2.6. Summary of Physiologically Relevant Pharmacological Targets

**Figure 5:**
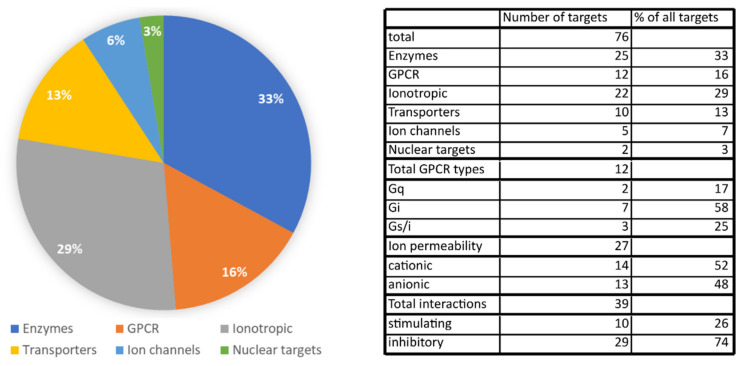
Pie chart showing the percentage of specific protein targets of CBD from a pool of all protein targets. Table presenting the number of identified CBD targets and percentage of specific Gα proteins coupled to GPCR receptors and ion permeability of both ion channels and ionotropic receptors. The total number of final effects on cell membrane potential was calculated based on the type of Gα proteins coupled to GPCR receptors targeted by CBD and ion permeability of those targets together with pharmacological action of CBD (for example, inhibition of anionic ion channel was counted as a stimulatory effect).

We identified a total of 76 different molecular targets of CBD. Among those, enzymes and ion channels/ionotropic receptors make up most of the targets. GPCRs and transporter proteins are less abundant targets of CBD, and there are only two different targets that are nuclear receptors. Among enzymes, CBD potently interacts with various CYP450 enzymes involved in drug metabolism, which raises concerns about possible drug interactions. Numerous targets among ion channels and ionotropic receptors might explain CBD’s efficacy as a seizure controlling drug, but they may also contribute to the anti-nociceptive action of CBD. On the other hand, targets among GPCRs and transporters represent a link between CBD’s actions and the endocannabinoid system because of CBD’s high activity for the FABP proteins and allosteric modulation of cannabinoid receptors. The contribution of various protein types (enzyme, receptor, ion channel, etc.) is presented in Figure 5. We also analyzed the number of possible interactions with transmembrane receptors in relation to possible outcomes on cell membrane potential and cellular activity. Receptors coupled with Gi protein or anion-permeable channels were assumed to be inhibitory, whereas cation-permeable channels together with GPCRs coupled with Gq and possibly Gs were assumed to be stimulatory. This analysis revealed that almost 75% of direct CBD–receptor interactions should inhibit cell activity, whereas only 26% of interactions were able to stimulate cellular activity. More of the pharmacological effects of CBD, including the modulation of cytokine factors, cell function and reactive oxygen species may be found in the appendix table in the paper by McPartland et al., 2014 [60].

## 3. The Analgesic Potential of CBD

Preclinical and clinical studies suggest a potential anti-nociceptive effect of CBD and CBD combined with other compounds in several pain-related diseases. Analgesic effects may vary depending on the dose and route of drug administration. The following section summarizes the newest findings in a various pain-related diseases and CBD effectiveness in pain management.

Several studies have reported that in healthy rodents submitted to a painful experience (e.g., tail-flick or paw pressure test) CBD administration may diminish the nociceptive experience. Moreover, depending on the experimental design, CBD may potentiate or antagonize Δ9-THC’s effect. Intra-ventrolateral periaqueductal grey (PAG) microinjections of CBD or cannabichromene (CBC) resulted in a dose-dependent reduction in the ongoing activity of ON and OFF neurons in anaesthetized rats and caused analgesia, measured by the tail-flick test (effects were antagonized by selective antagonists of CB1, adenosine A1 and TRPA1, but not TRPV1 receptors) [61]. In turn, regarding the effects of CBD on Δ9-THC, Britch et al. reached inconsistent conclusions. In their study, CBD alone had no anti-nociceptive effect, however, it did increase animals’ locomotor activity. When administered 15 min before Δ9-THC, CBD enhanced Δ9-THC-induced paw pressure, but not tail withdrawal, anti-nociception and enhanced rats’ hypolocomotion (at low Δ9-THC dose). However, when administered 13 h or 15 min before Δ9-THC (in the lowest tested dose 1.8 mg/kg), CBD (in the highest tested dose of 30 mg/kg) had no effect on Δ9-THC’s effect and inhibited Δ9-THC’s metabolism (this effect was more prominent in females than in males) [62]. Greene et al. demonstrated that chronic daily CBD administration enhanced the development of tolerance to Δ9-THC-induced anti-nociception, likely due to CBD-induced inhibition of Δ9-THC metabolism or due to antagonism of Δ9-THC’s effects after repeated treatment [63]. Cannabinoid-mediated neuromodulation may also be involved in transcutaneous electrical nerve stimulation (TENS)-mediated anti-nociception, a non-pharmacological therapy for the treatment of pain. In rats submitted to 10 or 150 Hz TENS, CBD and naloxone abolished the anti-nociceptive effects of both 10 and 150 Hz TENS [64].

### 3.1. Animal Models of Neuropathic Pain

Neuropathic pain arises from direct nerve damage or a disease and affects the somatosensory nervous system. Hyperalgesia, an excessive pain sensation, might be accompanied by allodynia, a painful sensation of neutral stimulus. Neuropathic pain is persistent and difficult to relieve; therefore, identification of novel, more effective therapeutic strategies is needed.

CBD exerts an analgesic effect in several neuropathic pain models. In a sciatic nerve injury mouse model, CBD-containing gelatine administered orally ad libitum significantly reduced allodynia up to 3 weeks post-surgery. Similar results were obtained in the Δ9-THC and morphine gelatine groups; however, tolerance to morphine developed after one week of treatment, which was not observed after CBD or Δ9-THC treatment. Moreover, the same study revealed no significant effect of CBD in hyperalgesia reduction, although an analgesic trend was observed [65]. In rats, after ligation of the L5 spinal nerve, CBD and its modified derivatives (dihydroxyl-CBD, and didesoxy-CBD) suppressed chronic neuropathic pain. The analgesic effect was correlated with cannabinoid potentiation of the α3 GlyRs but not with their binding affinity for CB1 and CB2 receptors. Furthermore, glycine currents in dorsal horn neurons in spinal cord slices from neuropathic rats were enhanced by cannabinoids [66]. In a mouse chronic neuropathic pain model involving foramen rotundum inflammatory constriction trigeminal infraorbital nerve injury, CBD administered orally in a peanut butter vehicle alleviated mechanical allodynia within 1 h and remained significant through 6 h [67]. CBD effectiveness was also demonstrated in a chronic constriction injury (CCI) animal model of neuropathic pain. Both oral and subcutaneous administration of CBD resulted in reduced surgery-induced mechanical and thermal allodynia [12,68]. Costa et al. also observed a reduction in the content of several pain mediators (prostaglandin E_2_ plasma concentration and paw tissue malondialdehyde, nitric oxide and glutathione-related enzymes levels) in response to 20 mg/kg oral administration of CBD. The anti-hyperalgesic effect was prevented by capsazepine (the vanilloid receptor antagonist) but not by rimonabant or SR144528 (CB1 and CB2 receptors antagonists, respectively) [12]. Δ9-THC has stronger effects on pain reduction than CBD; nevertheless, side effects exclude its clinical use. However, Δ9-THC and CBD co-administration provided a 200-fold increase in low dose efficacy, with no side effects observed (only high co-administered doses caused side effects, similar to those seen with Δ9-THC alone) [68]. After spinal cord injury, CBD may act as an anti-inflammatory agent. Li et al. have shown that intraperitoneal (i.p.) CBD injections attenuated pro-inflammatory cytokine and chemokine invasion and prevented thermal sensitivity development [69]. In turn, in a peripheral sciatic nerve cuff neuropathic pain rat model, CBD, as well as Δ9-THC and CBD + Δ9-THC co-administration, reduced hypersensitivity and promoted beneficial changes in myelinated Aβ mechanoreceptive fibers [70]. CBD also modulates serotoninergic transmission in dorsal raphe nucleus. Intravenous acute administration enhances the firing rate of 5-HT neurons in dorsal raphe nucleus of rats. This effect was blocked by 5HT1a and TRPV1 receptor antagonists (capsazepine and WAY100635, respectively). Repeated subcutaneous administration of CBD for 7 days enhances 5-HT firing through desensitization of 5HT1a receptors in healthy rats. In a spared nerve injury neuropathic pain rat model, repeated CBD injections diminished mechanical allodynia, anxiety-like behavior and normalized 5-HT activity. This effect was blocked by capsazepine and partially by WAY100635 [71].

In addition to surgically induced neuropathy, chronic pain can be triggered by chemical substance administration. For example, chemotherapeutic paclitaxel (PAC) might be responsible for peripheral neuropathy development in humans. In several studies of PAC-induced neuropathic pain, CBD exerted a positive effect against mechanical and thermal allodynia in mice and prevented mechanical sensitivity. The observed effect was reversed by the 5HT1a receptor antagonist WAY100635 but not by CB1 or CB2 receptor antagonists [72,73,74]. Furthermore, CBD and Δ9-THC co-administration enhanced the effect of low, ineffective doses of each drug alone [74]. In cisplatin-induced neuropathy, CBD reduced neuropathic pain in mice but did not prevent pain development, when administered 30 min prior to cisplatin [75]. Moreover, Pan et al. demonstrated that CBD prevents cisplatin-induced oxidative/nitrosative stress, inflammation and cell death in the kidney and improves renal function [11].

Type 1 diabetes is often associated with neuropathic pain development. In a rodent diabetes type 1 model, CBD delayed disease development and exerted a beneficial effect on pancreatic microcirculation in tested animals (reduction in leukocyte activation and increased functional capillary density) and attenuated myocardial dysfunction, cardiac fibrosis, oxidative/nitrosative stress, inflammation and cell death, while enhancing the ability of arteries to relax via enhanced production of vasodilator cyclooxygenase-1/2–derived products [50,76,77]. Intranasal and i.p. administration of CBD also attenuated neuropathic pain and inhibited elevation of microglial density and phosphorylated p38 mitogen-activated protein kinases [78]. Moreover, in streptozotocin-induced diabetic rats, acute i.p. CBD administration produced an anti-allodynic effect in the Von Frey test. This effect was mediated by serotoninergic system activation through 5HT1a receptors, since WAY100135, a selective 5HT1a receptor antagonist, but not CB1, CB2 or glycine antagonists, prevented the observed effect. When administered chronically, CBD reduced mechanical allodynia and prevented the decrease in serotonin levels in the spinal cord [79].

### 3.2. Inflammatory Pain

Inflammatory pain is caused by noxious stimuli that occur during the inflammatory or immune response. Under normal conditions, inflammation is a crucial protective mechanism, which plays an important role in the wound healing process. It is usually accompanied by redness, heat, swelling, pain/hypersensitivity, and loss of function. Nevertheless, in pathological conditions, inflammation may cause long-lasting pain through activation of sensory neurons [80].

In several inflammatory-induced chronic pain models, cannabinoids, including CBD, may exert an analgesic and anti-inflammatory effects. A study with complete Freund’s adjuvant (CFA)-induced inflammatory pain in rodents revealed an important role for CBD and its modified derivatives in chronic pain attenuation [66]. In two additional murine models of induced inflammation, using 2.5% Croton oil in acetone topically applied to the ear or by i.p. injection of lipopolysaccharide (LPS), Verrico et al. demonstrated that CBD diminished serum levels of pro-inflammatory factors interleukin 6 (IL-6) and tumor necrosis factor α (TNFα) and increases levels of the anti-inflammatory cytokine interleukin 10 (IL-10). Similar results Verrico et al. were obtained in an in vitro model of inflammation in the same study [81]. On the other hand, Britch et al. demonstrated that twice daily i.p. administration of Δ9-THC for 3 days caused reduction in pain-related behavior in a CFA-induced inflammatory pain model but had little or no effect on changes in serum cytokines levels or paw edema. In turn, CBD i.p. administration had minimal effects on inflammatory pain but significantly reduced interleukin 1β (IL-1β), IL-10, interferon γ (IFN-γ) levels and increased IL-6 levels [82]. This result suggests a more beneficial role for Δ9-THC compared to CBD in pain attenuation due to the ambiguous results in inflammatory-related factor levels in the serum. Similar conclusions may be drawn from the work of Karmaus et al., where orally delivered CBD in corn oil enhanced LPS-induced pulmonary inflammation, augmented inflammatory cell infiltrate in bronchoalveolar lavage fluid and enhanced pro-inflammatory cytokine mRNA levels (TNFα, IL-6, interleukin-23 (IL-23), granulocyte colony stimulating factor (GCSF)) [83]. In another study, inflammatory pain was induced by carrageenan injection, and the effect of cannabidiolic acid (CBDA), CBD and Δ9-THC were investigated. I.p. administration of CBDA prior to carrageenan produced dose-dependent anti-hyperalgesia and anti-inflammatory effects. Similar effects were obtained with oral CBDA and Δ9-THC administration. A CB1 receptor antagonist, rimonabant, blocked the analgesic effect of Δ9-THC, while CBDA’s effects were blocked by AMG9810 (a TRPV1 antagonist). Unfortunately, in this study, CBD did not reduce carrageenan-induced hyperalgesia. This result may suggest a more potent role for CBDA compared to CBD in inducing analgesic effects [84]. Moreno-Martet et al. investigated the role of Sativex-like (Sativex/Nabiximols is a cannabinoid drug containing 1:1 CBD and Δ9-THC) combined with a Δ9-tetrahydrocannabinol botanical drug substance (Δ9-THC-BDS) and cannabidiol-botanical drug substance (CBD-BDS) in an experimental autoimmune encephalitis (EAE) model of multiple sclerosis in mice. Results demonstrated that the Sativex-like combination and Δ9-THC-BDS alone improved neurological deficits in EAE animals and reduced the number and extent of cell aggregates in the spinal cord, derived from cell infiltration to the central nervous system. Rimonabant reversed the observed neurological benefits and reduced cell aggregates in the Δ9-THC-BDC-treated group. Notwithstanding, CBD-BDS alone did not provide these effects, merely delaying disease onset [85]. Moreover, CBD ameliorated the severity of experimental autoimmune encephalomyelitis in mice, diminishing axonal damage, inflammation and T-cell recruitment in the spinal cord of mice [10].

### 3.3. Arthritis-Related Pain

Osteoarthritis (OA) is a degenerative joint disease primarily affecting people over 60 years old. Due to the increased ageing population and increased rates of obesity, OA has become a serious, global problem. There is no cure for this disease, and current therapy includes maintaining joint functionality through rehabilitation and losing weight, with pain alleviation using nonsteroidal anti-inflammatory drugs (NSAIDs) and opioids in more severe cases.

In preclinical models, CBD exerts an analgesic effect via different routes of administration. In a spontaneous canine OA model, CBD increased dogs’ mobility and reduced pain, while no side effects were observed, Gamble et al. demonstrated increased alkaline phosphatase during CBD treatment [81,86]. In rodent OA models, disease is usually induced by intra-articular (i.a.) sodium monoiodoacetate (MIA) injection. In osteoarthritic rats, i.a. CBD administration dose-dependently decreased the joint afferent firing rate. Moreover, an increase in paw withdrawal threshold and weight bearing were observed. A TRPV1 antagonist, SB-366791, significantly inhibited the analgesic effect of CBD with respect to hind paw withdrawal threshold but did not have a significant effect on weight. CBD also reduced local inflammation by decreasing rolling and adherent leukocytes. The anti-rolling effect of CBD at 30 min was blocked by CB2 and TRPV1 antagonists but not a CB1 antagonist. The anti-adherence effect was blocked only by SB-366791. Prophylactic i.a. CBD administration prevented joint pain development and exerted a neuroprotective effect [87]. In a rat complete Freund’s adjuvant-induced monoarthritic knee joint model, transdermal CBD gel applied for 4 days dose-dependently reduced joint swelling, limb posture scores, synovial membrane thickening and animals’ pain. Furthermore, pro-inflammatory factors (Calcitonin gene-related peptide or OX42) were reduced in spinal cord and dorsal root ganglia (TNFα) [9]. In a murine collagen type II-induced arthritis, daily i.p. or oral CBD administration reduced disease progression and blocked LPS-induced increases in serum TNFα. In vitro culture of synovial cells collected from mice treated with CBD released decreased TNFα compared to cells collected from control animals. In vitro CBD also inhibited the release of reactive oxygen species by Zymosan-stimulated neutrophils [88]. The latest study by Lowin et al. showed that in human in vitro culture of rheumatoid arthritis synovial fibroblasts, CBD increases intracellular calcium levels, reduces cell viability, and IL-6/interleukin 8 (IL-8)/MMP-3 production. A TRPA1, but not TRPV1, antagonist reduced the effects of CBD. The effect was enhanced by TNFα pretreatment, which may suggest that CBD preferentially targets pro-inflammatory (activated) synovial fibroblasts, suggesting potential anti-arthritic activity [89]. Similar results from the work of Winklmayr et al. in an in vitro human chondrocytes culture showed that CBD concentrations greater than 4 μM diminished cell viability and increased caspase 3/7 activity, elevated Ca^2+^ and Extracellular signal-regulated kinases 1/2 phosphorylation and enhanced apoptotic cell population. This effect was mediated via the CB1 receptor, since AM251 inhibited Ca^2+^ influx and reduced the toxic effects of CBD [90].

### 3.4. Other Pain Models

Individual studies indicate a potential beneficial role for CBD in various disease animal models. Systemic (i.p.) injection of CBD reduced mechanical allodynia and reversed conditional place preference produced by peripheral nerve block in a rat model of incision pain. Moreover, direct rostral anterior cingulate cortex injection of CBD produced a similar, dose-dependent effect [91]. In a rat model of myofascial pain (induced by nerve growth factor intramuscular injection), intramuscular injection of CBD or cannabinol (CBN) decreased mechanical sensitization and increased the mechanical threshold of masseter muscle mechanoreceptors [92]. CBD may also enhance morphine-induced anti-nociception. In animal models in which n-methyl-d-aspartate (NMDA) receptor over activity plays a crucial role (opioid analgesia attenuation, NMDA-induced convulsive syndrome and ischemic stroke), intracerebroventricular (icv) injection of CBD enhanced morphine-evoked supraspinal anti-nociception, mitigated NMDA-induced convulsive syndrome and reduced the infarct size caused by permanent unilateral middle cerebral artery occlusion. This effect was absent in sigma-1 receptor (σ1R) knock-out mice and was reduced by the σ1R agonists PRE084 and PPCC [93]. Neelakantan et al. demonstrated that distinct mechanisms of action may underlie the interactions between CBD and morphine in the different behavioral assays. Morphine alone diminished nociceptive responses in all three tested pain models (acetic acid-stimulated stretching, acetic acid-decreased operant responding for palatable food and hot plate thermal nociception), whereas CBD alone produced an anti-nociceptive effect only in the acetic acid-stimulated stretching model. Nevertheless, CBD and morphine combination produced synergistic effects in reversing acetic acid-stimulated stretching behavior, but subadditive effects in the hot plate thermal nociceptive assay and the acetic acid-decreased operant responding for palatable food assay [94]. In a 6-hydroxydopamine-induced Parkinson’s disease mouse model, CBD exerted analgesic effects by increasing anandamide binding to the CB1 and TRPV1 receptors. URB597, a relatively selective FAAH inhibitor, potentiated the CBD effect, blockade of CB1 (by AM251) inhibited CBD effect, and TRPV1 receptor antagonism by capsazepine increased the anti-nociceptive effect of CBD [95]. In a corneal hyperalgesia mouse model, CBD produced anti-nociceptive effects and reduced neutrophil-infiltration in a wild-type and CB2 knock-out mice. The effect of CBD was blocked by a 5HT1a receptor antagonist, WAY100635, in wild-type mice, indicating the involvement of 5-HT1a and CB2 receptors in analgesic and anti-inflammatory actions [8]. However, Finn et al. proved that CBD alone did not reduce nociception in a rat model of persistent pain, in contrast to Δ9-THC, morphine and its combinations, and did not modulate Δ9-THC’ anti-nociceptive effects [96].

### 3.5. Clinical Studies

The positive effects of CBD were confirmed many years ago [97,98,99], encouraging clinical trials. CBD was proven to be safe and cause only mild adverse effects in humans (e.g., ataxia, sedation, nausea, headache or decreased appetite) [100,101,102,103]. A transdermal CBD-containing gel in patients with peripheral neuropathic pain mitigated pain, as well as cold and itchy sensations [104]. CBD might be used clinically alone or in combination with other cannabinoids. Epidiolex, a pure CBD containing drug, is indicated for the treatment of seizures associated with Lennox Gastaut syndrome, Dravet syndrome, or tuberous sclerosis complex in patients 1 year of age and older. In treatment-resistant epilepsy, CBD reduced seizure frequency in both children and adults [105]. In Lennox-Gestaut syndrome, a rare and severe childhood-onset form of epilepsy, oral CBD reduced seizure frequency and improved patients’ overall conditions but triggered adverse effects (somnolence, decreased appetite, diarrhea; more severe in higher doses groups) [106,107]. CBD has shown similar results for the treatment of Dravet syndrome, a drug-resistant epilepsy that begins during the first year of life. CBD diminished major motor seizures and improved patients’ overall condition [108,109].

In a study of chronic pain patients by Caparo et al., gels containing 15.7 mg CBD, 0.5 mg Δ9-THC, 0.3 mg cannabidivarin, 0.9 mg cannabidiolic acid, 0.8 mg cannabichrome and >1% botanical terpene blend caused reduced opioid use (in 53% of patients) and improved quality of life and sleep after 8 weeks of cannabinoid treatment [110]. However, in a study investigating three CBD- and Δ9-THC-containing drugs (Bedrocan 22.4 mg Δ9-THC + <1 mg CBD; Bediol 13.4 mg Δ9-THC + 17.8 mg CBD; Bedrolite 18.4 mg CBD + <1 mg Δ9-THC), none of the treatments had an effect greater than placebo on spontaneous or electrical pain responses. Drugs containing Δ9-THC increased the pressure pain threshold, while CBD increased Δ9-THC plasma concentrations [111].

The majority of clinical studies describe the efficacy of CBD and Δ9-THC co-administration, generally in doses of 2.5 mg CBD and 2.7 mg Δ9-THC in an oral mucosa spray, with treatment periods varying from one to several weeks. After treatment sessions, patients reported reduced pain, improved sleep quality, and reduced insomnia and fatigue [112,113,114,115]. One of the best studied CBD-containing registered products is Sativex^®^ (Nabiximols), containing the abovementioned doses of CBD and Δ9-THC. Clinical studies have demonstrated its efficacy in pain attenuation in several diseases. It was proven to mitigate neuropathic pain and improve quality of life in patients suffering from several diseases, e.g., multiple sclerosis, cancer or rheumatoid arthritis [116,117,118,119]. Lichtman et al. proved that U.S. patients (but not patients from the rest of the world) experienced significant benefits from Nabiximols in cancer-related attenuation of pain. This effect might be due to lower doses of opioids taken at baseline or different distribution of cancer pain types [120]. In turn, Lynch et al. did not observe a significant reduction in pain intensity in chemotherapy-induced neuropathic pain compared to placebo. However, five of 16 patients reported a two-point or greater reduction in pain [121]. A study by Santoro et al. showed that in multiple sclerosis patients, treated or not with interferon-1β (IFN-1β), Sativex^®^ decreased *CNR2* expression levels in peripheral blood mononuclear cells (this effect was not observed in patients who were not treated with IFN-1β during the study) [122].

## 4. Summary

CBD is a well-tolerated and safe natural compound exerting analgesic effects in various animal models of pain, as well as clinical studies. The referenced studies indicate a positive influence of CBD in treatment for various diseases in both pre-clinical and clinical trials. In the majority of animal studies, CBD has been demonstrated to exert analgesic effects, diminishing hyperalgesia and mechanical/thermal allodynia through various routes of administration. When co-administered with Δ9-THC, CBD may reduce the effective dose and diminish negative side effects of Δ9-THC [68,74]. However, some studies indicate no modulation of Δ9-THC’s effects by CBD [96]. Moreover, CBD has the potential to act in an anti-inflammatory way; however; the effects are unclear. In the inflammatory-related pain modes, some research gives hope for an anti-inflammatory action of CBD, while others have reported opposing results. More detailed research is needed to clarify this effect; however, the inflammatory state is not always negative. In arthritic in vitro studies, CBD promoted chondrocyte and synoviocyte apoptosis but with stronger effects on the inflammatory-activated cells, which may be a positive result that indicates anti-arthritic activity [89,90]. The discussed studies indicate a positive influence of CBD on various diseases; nevertheless, animal studies cannot always be translated into human results. It is also important to remember that there are few to no studies on chronic CBD administration in healthy people. Despite the fact that there are trials on heathy volunteers that demonstrate CBD’s safety and good tolerance, these studies do not examine prolonged use, which is common in chronically ill people. Patients suffering from chronic pain are often forced to take medications continuously for many years; it is not possible to test the effects of CBD on healthy people for such a long time [123,124]. Animal studies with chronic CBD treatment last several weeks, up to several months, which may reflect the prolonged use in people, but the differences could not be excluded. In one animal study, Ignatowska-Jankowska et al. demonstrated that repeated CBD treatment may inhibit specific immunity by reducing T, B, T cytotoxic, and T helper cell numbers, while increasing the number of NK and NKT cells involved in nonspecific antiviral and antitumor immune response [125]. Chronic CBD treatment allows for long-term therapeutic effects to be achieved, without significant side effects or tolerance development. Especially important are prolonged anti-inflammatory effects, which may have an influence more directly on the cause of the pain development and therefore provide long-lasting analgesic effect. Acute CBD treatment may not be sufficient to combat the cause of the pain and provide an effect lasting up to several hours (depending on the disease model, dose and route of administration).

CBD is a substance of great therapeutic potential but is currently very understudied. Human CBD short-term studies illustrate low toxicity and mild adverse effects. However, it is unknown what effects will be caused by “CBD gummies” or “CBD water”, which are increasingly offered by manufactures to a wide audience. CBD’s pharmacology is not as clear as Δ9-THC, where the exact mechanism of action is known. Here, we still have much to learn.

Due to its complex pharmacological profile, effects observed from CBD administration vary and might be state-dependent. In vivo studies have revealed significant roles of various molecular targets in different models, namely, 5HT1a, CB1, CB2, TRPV1, α3 GlyRs, adenosine A1 and TRPA1. These targets were often not replicated throughout various models, suggesting the hypothesis of state-dependent effects of CBD. To further support this hypothesis, we emphasize CBD interactions with the endocannabinoid system. CBD may act as an indirect agonist of cannabinoid receptors through increased endocannabinoid tone [95], most likely through FABP inhibition [43,44], but on the other hand, it can directly antagonize the CB1 receptor [14]. Similarly, CBD activity for the CB2 receptor is very complex, including both partial agonism and negative allosteric modulation [14]. To complicate the issue even further, it is known that partial agonist action is dependent on receptor expression, density, and tonic activity of the system; therefore, it may vary in different tissues and under different conditions [126], further supporting our state-dependent hypothesis. Unsurprisingly, many of the abovementioned targets include ion channels, such as TRPV1 and TRPA1 receptors or α3 GlyRs. Furthermore, the role of adenosine receptors in the anti-inflammatory and anti-nociceptive effects of CBD is worth further study as CBD is a potent inhibitor of adenosine reuptake; therefore, adenosine receptors might be the node of CBD’s action. Moreover, it would be interesting to see the role of other molecular targets of CBD involved in pain transmission, particularly dopamine D2 receptors or GPRs among GPCRs and, more importantly, various ionotropic targets, such as calcium or sodium channels, GABA receptors, 5HT3A or α7nACh receptors.

Compared to Δ9-THC, CBD has fewer unwanted side effects, and they are milder. These results provide hope for successful CBD use in the clinic in the future; however, more studies are required for precise elucidation of CBD’s mechanisms of action. We hope that this review, together with data tables regarding CBD’s pharmacology, may facilitate the understanding of CBD’s potential in various pain conditions. Importantly, serious consideration should be applied to possible drug–drug interactions due to a significant proportion of CBD’s interaction with cytochrome P450 isoforms.

## Supplementary Materials

Supplementary materials can be found at https://www.mdpi.com/1422-0067/21/22/8870/s1. Materials include a table summarizing results regarding CBD’s action in pain research and a CSV table containing CBD’s pharmacological data. If you are willing to use the tables for further analysis, please cite this paper.

## Abbreviations

Δ9-THC	Δ^9^tetrahydrocannabinol
Δ9-THC-BDS	Δ9-tetrahydrocannabinol botanical drug substance
σ1R	Sigma-1 receptor
2-AG	2-arachidonoylglycerol
5HT1a	Serotonin 1A receptor
AANAT	Aralkylamine N-acetyltransferase
ABCC1	Multidrug resistance-associated protein 1
ABCG2	ATP-binding cassette super-family G member 2
ACAT	Acyl-CoA cholesterin acyltransferase
AEA	Anandamide
CAMP	Cyclic adenosine monophosphate
CB	Cannabinoid receptor
CBD	Cannabidiol
CBDA	Cannabidiolic acid
CBD–BDS	Cannabidiol-botanical drug substance
CBC	Cannabichromene
CBN	Cannabinol
CCI	Chronic constriction injury
CFA	Complete Freund’s adjuvant
CHO	Chinese hamster ovary cells
CYPs	Cytochrome P450 superfamily
DOR	δ-opioid receptor
EAE	Experimental autoimmune encephalitis
ENT	Equilibrative nucleoside transporter
EROD	Ethoxyresorufin-O-deethylase
FAAH	Fatty acid amide hydrolase
GCSF	Granulocyte colony stimulating factor
GlyRs	Glycine receptors
GPCR	G-protein coupled receptor
GPR	Orphan G-protein coupled receptor
GTP	Guanosine 5′-O-[gamma-thio]triphosphate
HEK293	Human embryonic kidney 293 cells
HPLC	High-performance liquid chromatography
HTRF	Homogeneous Time Resolved Fluorescence
i.a.	Intra-articular
IDO	Indoleamine-pyrrole 2,3-dioxygenase
IFN-γ	Interferon- γ
IFN-1β	Interferon-1β
IL	Interleukin
i.p.	Intra-peritoneal
LOXs	Lipooxygenases
LPS	Lipopolysaccharide
MIA	Sodium monoiodoacetate
MOR	*μ*-opioid receptor
NAM	Negative allosteric modulator
NBD-stearate	12-*N*-methyl-(7-nitrobenz-2-oxa-1,3-diazo)aminostearic acid
NMDA	*N*-methyl-d-aspartate receptor
NSAIDs	Nonsteroidal anti-inflammatory drugs
OA	Osteoarthritis
PAC	Paclitaxel
PAG	Periaqueductal grey
PAM	Positive allosteric modulators
PBMC	Peripheral blood mononuclear cell
PLA2	Phospholipase A2
PPRγ	Peroxisome proliferator-activated receptor gamma
TENS	Transcutaneous electrical nerve stimulation
TNFα	Tumor necrosis factor α
TRP	Transient receptor potential channel
VDAC1	Voltage-dependent anion channel 1
