# Supplementary material for: Cannabidiol for Pain Treatment: Focus on Pharmacology and Mechanism of Action
Source: Int J Mol Sci. 2020 Nov 23;21(22):8870. doi: 10.3390/ijms21228870 (PMC7700528; doi:10.3390/ijms21228870)
Supplement: Supplementary file 1 [file ijms-21-08870-s001.pdf]

No	Model	Dose/route of administration	Key findings	Ref.
**Healthy rodents submitted to painful experience**				
1	Male Wistar Rats submitted to tail flick test and measurement of the electrical activity of anaesthetizes rat's ON and OFF neurons of the rostral ventromedial medulla	intra-vl-PAG microinjection of vehicle; CBD (1.5; 3; 6 nmol) alone or CBD (3 nmol) in combination with the selective antagonists of TRPV1 (I-RTX, 1 nmol), CB1 (AM251, 0.5 nmol), adenosine A1 (DPCPX, 0.05 nmol), TRPA1 (AP18, 6 nmol), 5-HT1A (WAY100635, 0.34 nmol); CBC (3; 6 nmol) alone or CBC (6 nmol) in combination with I-RTX (1 nmol), AM251 (0.5 nmol), DPCPX (0.05 nmol) or AP18 (6 nmol); OMDM-2 (selective inhibitor of endocannabinoid cellular reuptake, 1.5; 3 nmol) or mustard oil (TRPA1 channels agonist, 3; 6 nmol)	CBD and CBC: ↓ ongoing activity of ON and OFF neurons (dose-dependently); ↑ antinociceptive responses in the tail flick-test (effects antagonized by selective antagonists of CB1 adenosine A1 and TRPA1, but not of TRPV1 receptors); ↑ endocannabinoid levels in the ventrolateral periaqueductal grey<br>OMDM-2 and mustard oil: similar effects	(Maione et al. 2011)
2	Male and female Sprague-Dawley rats tested on tail withdrawal, paw pressure and locomotor activity tests	Experiment 1: CBD (0, 10, 30 mg/kg) 15 min before Δ9-THC (0, 1.8, 3.2, 5.6, 10 mg/kg); test 15–360 min post-Δ9-THC injection<br>Experiment 2: CBD (30 mg/kg) 13 h or 15 min before Δ9-THC (1.8 mg/kg); test 30–480 min post-Δ9-THC injection<br>Experiment 3: CBD (30 mg/kg) 13 h or 15 min before Δ9-THC (1.8 mg/kg); serum samples taken 30–360 min post-Δ9-THC injection	Experiment 1:<br>CBD alone: no antinociceptive effects; ↑ locomotor activity<br>CBD+Δ9-THC: ↑ THC-induced paw pressure but not tail withdrawal antinociception; ↑ Δ9-THC-induced hypolocomotion at lower Δ9-THC doses<br>No sex differences in CBD-Δ9-THC interactions<br>Experiments 2 and 3:<br>CBD: no enhancement of Δ9-THC's effects; ↓ THC metabolism (effect greater in females than males)	(Britch et al. 2017)

3	Male and female Sprague-Dawley rats submitted to tail withdrawal and paw pressure tests	Twice-daily treatment with vehicle, CBD (10 mg/kg), Δ9-THC (3.6 mg/kg females; 9.3 mg/kg males) or CBD+Δ9-THC for 4 days	Δ9-THC day 1: more potent in females than in males (both nociceptive tests)<br><br>Δ9-THC days 1-6: ↓ of potency on the tail withdrawal test (more in females than males)<br><br>CBD+Δ9-THC days 1-6: greater rightward/downward shifts of the Δ9-THC dose-effect curve than Δ9-THC alone<br><br>Day 6 blood samples: higher serum Δ9-THC levels in CBD+Δ9-THC-treated females than in vehicle + Δ9-THC-treated females; Δ9-THC's active metabolite 11-OH- Δ9-THC and its inactive metabolite Δ9-THC-COOH: lower in CBD + Δ9-THC-treated rats than in vehicle+ Δ9-THC-treated rats (both sexes); CBD: ↑ serum levels of the active metabolite cannabinol (both sexes)	(Greene et al. 2018)
4	Male Wistar Rats submitted to transcutaneous electrical nerve stimulation (TENS) and the tail-flick test	Naloxone (3.0 mg/kg), diazepam (1.5 mg/kg) or CBD (0.75 mg/kg,1.5 mg/kg, 3.0 mg/kg, 4.5 mg/kg, 6.0 mg/kg and 12.0 mg/kg) i.p. 10 min after the acute administration,10 Hz or 150 Hz TENS was performed for 30 min	Naloxone and CBD: ↓ nociceptive threshold; ↓ tail-flick latencies<br><br>Diazepam: no alterations in the nociceptive threshold	(Gonçalves et al. 2014)
**Neuropathic pain**				
5	Sciatic nerve injury mouse model	Gelatin oral self-administration paradigm - mice consumed Δ9-THC-, CBD-, or morphine-containing gelatins *ad libitum* for 3 weeks	Δ9-THC gelatin: ↓ allodynia; ↓ hyperalgesia; ↓ ultrasonic vocalizations<br><br>CBD gelatin: ↓ allodynia; ↓ ultrasonic vocalizations	(Abraham et al. 2020)

			Morphine gelatin: ↓ allodynia (tolerance after 1 week); ↓ ultrasonic vocalizations	
6	Ligation of L5 spinal nerve neuropathic pain model in rats	CBD (3, 10, 50 ug i.t.)<br>Dihydroxyl-CBD, DH-CBD (10, 30, 50 mg/kg i.p.)<br>Didesoxy-CBD, DD-CBD (0.1, 1, 10, 15 mg/kg i.p.)<br>Antagonist tested: strychnine 1 μM (GlyR antagonist)	CBD and its modified derivatives: ↓ chronic neuropathic pain (positively correlated with cannabinoid potentiation of the α3 GlyRs, but not with their binding affinity for CB1 and CB2 receptors); ↑ glycine currents in dorsal horn neurons in rat spinal cord slices	(Xiong et al. 2012)
7	Foramen Rotundum Inflammatory Constriction Trigeminal Infraorbital Nerve injury mouse model	orally - 5 uL of whole plant extracted hemp oil combined with a peanut butter vehicle (0.138 mg/kg)	CBD: ↓ mechanical allodynia within 1 h with a peak reversal effect at 4 h; remained significant throughout the 6 h	(Vigil et al. 2020)
8	Sciatic nerve chronic constriction	Oral administration of CBD (2.5–20 mg/kg) daily from day 7 to 14 after the injury<br>Antagonists tested: capsazepine, rimonabant, SR144528	CBD in neuropathic pain model: ↓ hyperalgesia to thermal and mechanical stimuli; the 20 mg/kg dose normalized PGE_2 plasma concentration and paw tissue malondialdehyde, NO and glutathione-related enzymes levels<br>Vanilloid antagonist capsazepine: prevention of anti-hyperalgesic effect<br>Cannabinoid receptor antagonists (rimonabant and SR144528): no effect on the withdrawal threshold	(Costa et al. 2007)

9	Mouse chronic constriction injury (CCI) model of neuropathic pain	Subcutaneous Δ9THC, CBD and Δ9-THC:CBD co-administration in doses: 0.01-56 mg/kg<br><br>Antagonists tested: AM281, AM630 (3 mg/kg)	Δ9-THC: ↓ mechanical and cold allodynia; ↑ motor incoordination, catalepsy, sedation (dose-dependently)<br><br>CBD: ↓ allodynia (dose-dependently, smaller effect than Δ9-THC); no side effects<br><br>Δ9-THC:CBD co-administration: ↓ allodynia (dose-dependently); 200-fold increase in anti-allodynic potency (at low doses); only high doses gave side effects (similar to Δ9-THC alone)<br><br>AM281 (CB1 receptor antagonist): abolition of the THC effect on mechanical allodynia and cold allodynia (only parital)	(Casey, Atwal, and Vaughan 2017)
10	Spinal cord injury mouse model	CBD 1,5 mg/kg i.p. for 10 weeks	CBD: ↓ pro-inflammatory cytokines and chemokines; ↓ moderate to severe thermal sensitivity development (compared to vehicle group)	(Li et al. 2018)
11	Peripheral sciatic nerve cuff (surgically induced neuropathic pain rat model)	Orally, in medium chain triglyceride oil, for 14 days<br><br>CBD - 25 mg/60 kg per day<br><br>Δ9-THC - 5 mg/60 kg per day<br><br>CBD: Δ9-THC - 12,5:12,5 mg/60 kg per day	All groups: ↓ hypersensitivity in male rats (particularly CBD: Δ9-THC combination); ↑ beneficial changes in myelinated Aβ mechanoreceptive fibers	(Linher-Melville et al. 2020)
12	Naive rats and Spared nerve injury neuropathic pain rat model	1. Intravenous (i.v.) increasing doses of CBD (0.1-1.0 mg/kg) - naive rats only<br><br>Antagonists tested: WAY 100635 (300 mg/kg), AM 251 (1 mg/kg), capsazepine (1 mg/kg) i.v.	Naïve rats: acute CBD ↓ the firing rate of 5-HT neurons in dorsal raphe nucleus (blocked by WAY100635, 5HT1A antagonist and capsazepine, the TRPV1 antagonist); repeated CBD ↑ 5-HT firing through desensitization of 5-HT1A receptors	(De Gregorio et al. 2019)

		2. Repeated treatment with CBD (5 mg/kg/day, subcutaneously, for 7 days - naive and SNI rats<br><br>Antagonists tested: WAY (2 mg/kg, capsazepine (10 mg/kg) subcutaneously	SNI rats: repeated CBD ↓ mechanical allodynia (blocked by capsazepine and partially by WAY100635), anxiety-like behavior (blocked by WAY100635); normalized 5-HT activity	
13	Paclitaxel (PAC)-induced allodynia mouse model	CBD 5 or 10 mg/kg i.p.	CBD: prevention of cold and mechanical allodynia development	(Ward et al. 2011)
14	PAC-induced neuropathic pain mouse model	CBD pretreatment (2.5, 5.0 mg/kg, i.p.)<br><br>Antagonists tested: SR141716A (CB1), SR144528 (CB2), WAY100635 (5HT-1A) 5 mg/kg i.p.	CBD: PAC-induced mechanical sensitivity prevention (effect reversed by the 5-HT1A antagonist, but not the CB1 or CB2 antagonists)<br><br>CBD + PAC: additive to synergistic inhibition of breast cancer cell viability	(Ward et al. 2014)
15	PAC, oxaliplatin or vincristine-induced neuropathic pain mouse model	Paclitaxel-treated mice pretreated with CBD (0.625–20.0 mg/kg i.p.), Δ9-THC (0.625–20.0 mg/kg i.p.) or CBD + Δ9-THC (0.04 + 0.04–20.0 + 20.0 mg/kg i.p.)<br><br>Oxaliplatin/vincristine-treated mice pretreated with CBD (1.25–10.0 mg/kg i.p.), Δ9-THC (10.0 mg/kg i.p.) or Δ9-THC + CBD (0.16 mg/kg THC + 0.16 mg/kg CBD i.p.)	CBD alone: ↓ mechanical allodynia in PAC- and oxaliplatin-treated mice<br><br>Δ9-THC alone: ↓ mechanical allodynia in PAC- and vincristine-treated mice<br><br>CBD + Δ9-THC: synergistic effect of low, ineffective doses	(King et al. 2017)
16	Cisplatin-induced neuropathy mouse model	Experiment 1: after 8 daily administrations of cisplatin (2,3 mg/kg): vehicle, 100 mg/kg gabapentin, 2 mg/kg Δ9-THC, or 2 mg/kg CBD i.p.	Experiment 1: ↓ neuropathy in all groups<br><br>Experiment 2: no prevention of neuropathy development	(Harris et al. 2016)

		Experiment 2: CBD (0.0, 0.5, 1.0, and 2.0 mg/kg) or Δ9-THC (0.0, 0.5, 1.0, and 2.0 mg/kg) i.p. 30 min prior to cisplatin administration		
17	Cisplatin-induced nephropathy mouse model	CBD 2.5 to 10 mg/kg i.p. every day, starting 1.5 h before the cisplatin exposure	CBD: ↓ induced oxidative/nitrosative stress, inflammation, cell death in the kidney; ↑ renal function	(Pan et al. 2009)
18	Murine type I diabetic peripheral neuropathic pain model	CBD: 0.1; 1; 2 mg/kg intranasal (i.n.) or 1; 10; 20 mg/kg i.p.<br><br>Nabilone (nonselective CB1 and CB2 agonist): 0.01; 0.03; 0.06 mg/kg i.n. or 0.01; 0.3; 0.6 mg/kg i.p.<br><br>WIN55212-2 (CB1 agonist): 0.01; 0.03; 0.06 mg/kg i.n. or 0.01; 0.3; 0.6 mg/kg i.p.<br><br>SR144528 (CB2 antagonist): 0.01; 0.1; 0.2 mg/kg i.n. or 0.1; 1; 2 mg/kg i.p.<br><br>SR141716A (CB1 antagonist): 0.01 mg/kg; 0.1 mg/kg; 0.2 mg/kg i.n. or 0.1; 1; 2 mg/kg i.p.	Moderate-high doses of intranasal and i.p. CBD: ↓ neuropathic pain; ↓ microglial density in the dorsal spinal cord and phosphorylated p38 MAPK<br><br>CB1 and CB2 agonists: ↓ nociception, no effect on microglial density in the dorsal spinal cord and phosphorylation of p38 MAPK<br><br>CB1 or CB2 antagonists: no pronociceptive effects itself	(Toth et al. 2010)
19	Spontaneous non-obese type 1 diabetes mouse model	Daily 5 mg/kg CBD or control vehicle i.p. five times weekly for ten weeks	CBD: delay in type 1 diabetes development; ↓ leukocyte activation; ↑ functional capillary density in the pancreatic microcirculation	(Lehmann et al. 2016)
20	Streptozotocin-induced diabetic	CBD (0.1, 0.3 or 3 mg/kg, i.p.) acutely or subchronically (for 14 days)	Acute CBD (0.3 and 3 mg/kg): anti-allodynic effect	(Jesus et al. 2019)

	neuropathy model in rats	Antagonists tested: AM251 (CB1 receptor antagonist) 1mg/kg i.p.; AM630 (CB2 receptor antagonist) 1 mg/kg i.p.; strychnine hydrochloride (glycine receptor antagonist) 10µg/20µL i.t.; WAY100135 (serotonin receptor 5-HT1A antagonist) 10µg/20µL i.t.	CB1, CB2 receptor or glycine receptor antagonists pretreatment (AM251, AM630, strychnine): no alteration in the antinociceptive effect of CBD (3 mg/kg); WAY100135 pretreatment: prevention of the antinociceptive effect of CBD (3 mg/kg)<br><br>Subchronic CBD (0.3 or 3 mg/kg): ↓ mechanical allodynia; prevented the lowering of spinal cord level of serotonin in diabetic rats (0.3 mg/kg dose)	
**Inflammatory pain**				
21	Inflammatory pain models:<br><br>1. Intraplantar injection of 10 µl Complete Freund's adjuvant (CFA_ to the left hind paw<br><br>2. CFA suspended in an oil/saline injected s.c. into the plantar surface of the left hind paw	CBD: 3, 10, 50 ug i.t.<br><br>Dihydroxyl-CBD (DH-CBD): 10, 30, 50 mg/kg i.p.<br><br>Didesoxy-CBD (DD-CBD): 0.1, 1, 10, 50 mg/kg i.p. or 0.1, 10 µg i.t.<br><br>Antagonist tested: strychnine 1 µM (GlyR antagonist)	CBD and its modified derivatives: ↓ chronic inflammatory pain (positively correlated with cannabinoid potentiation of the alfa3 GlyRs, but not with their binding affinity for CB1 and CB2 receptors); ↑ glycine currents in dorsal horn neurons in rat spinal cord slices<br><br>Strychnine (GlyR antagonist): abolished IGly and the potentiation of DH-CBD<br><br>DD-CBD (i.t. or i.p.): antagonized the DH-CBD-induced analgesic effect (dose-dependently)	(Xiong et al. 2012)
22	CFA intraplantar injection	Oral administration of CBD (20 mg/kg) daily from day 7 to 14 after the intraplantar injection	CBD in neuropathic pain model: ↓ hyperalgesia to thermal and mechanical stimuli; ↓ PGE_2, NO, glutathione-related enzymes	(Costa et al. 2007)

	inflammatory pain in rats		Capsazepine (vanilloid receptor antagonist), but not cannabinoid receptor antagonists (rimonabant and SR144528): prevented the anti-hyperalgesic effect of CBD	
23	CFA-induced inflammatory pain	Δ9-THC (0.0–4.0 mg/kg, i.p.) or CBD (0.0–10 mg/kg, i.p.) twice daily for 3 days	Δ9-THC: ↓ pain-related behavior; no effect on hind paw edema; little tolerance developed; little to no change in serum cytokines<br>CBD: ↓ IL-1β, IL-10, IFN-γ; ↑ IL-6; minimal effect on inflammatory pain	(Britch et al. 2020)
24	a. Two murine models of induced inflammation (100 uL of 2,5 % Croton oil in acetone topically applied to the right ear or 200 ng LPS i.p.)<br>b. *In vitro* models: mouse monocyte cell line; human monocyte cell line; primary mouse PBMC; primary human PBMC cell lines SEB or LPS pre-treated	a. Croton oil model: 100 uL of 10 mg/mL CBD<br>LPS model: 1, 10, or 100 ug CBD i.p. or either CBD (100 mg) or 18.3% methyl salicylate/16% menthol topically at the LPS injection site<br>b. 100 ng/ml CBD	CBD in both models: ↓ proinflammatory cytokines IL-6 and TNF-α; ↑ anti-inflammatory IL-10	(Verrico et al. 2020)

25	LPS-induced pulmonary inflammation in mice	75 mg/kg CBD in corn oil (CO) by oral gavage (delivered 0.1 ml/10 g body weight) or CO (vehicle) for 3 days	CBD: ↑ pro-inflammatory cytokine mRNA production (TNFα, IL-6, IL-23, Gcsf)	(Karmaus et al. 2013)
26	Carrageenan-induced acute inflammation in the rat hind paw	I.p. pre-carrageenan: VEH or CBDA (10, 1000 μg/kg)<br>Oral pre-carrageenan group: VEH; CBDA (0.1, 1, 10, 100, 1000 μg/kg); Δ9-THC (100, 1000 μg/kg); or CBD (100, 10,000 μg/kg)<br>I.p. post-carrageenan group: CBDA (10, 1000 μg/kg) or VEH 60 min after carrageenan<br>Antagonist tested: rimonabant (a CB1 antagonist); AMG9810 (a TRPV1 antagonist) 1 mg/kg i.p.	CBDA i.p. 60 min prior to carrageenan (but not 60 min after carrageenan):↓ hyperalgesia and inflammation<br>Δ9-THC orally 60 min prior to carrageenan: ↓ hyperalgesia and inflammation<br>CBDA orally 60 min prior to carrageenan: ↓ hyperalgesia<br>CBD orally 60 min prior to carrageenan: no effect<br>Combined low doses of CBDA and Δ9-THC: ↓ hyperalgesia and inflammation<br>The effects of Δ9-THC were blocked by rimonabant, while CBDA's effects were blocked by AMG9810	(Rock, Limebeer, and Parker 2018)
27	Autoimmune encephalitis (EAE) model of Multiple Sclerosis in mice	Sativex-like combination of Δ9-THC-BDS (10 mg/kg) and CBD-BDS (10 mg/kg) with Δ9-THC-BDS (20 mg/kg) or CBD-BDS (20 mg/k i.p.<br>(BDS – botanical drug substance)<br>Antagonists tested: rimonabant (CB1 antagonist); T0070907 (PAR-γ inhibitor) 5 mg/kg	Sativex-like combination and Δ9-THC-BDS alone: ↓ neurological deficits; ↓ number and extent of cell aggregates in the spinal cord (derived from cell infiltration to the CNS)<br>CBD-BDS alone: disease delay only<br>Rimonabant: reversion of neurological benefits and reduction of cell aggregates in Δ9-THC-BDC-treated group<br>T0070907: no effect	(Moreno-Martet et al. 2015)

28	Autoimmune encephalomyelitis (EAE) induced by myelinoligodendrocyte glycoprotein (MOG) in mice	CBD: 5 mg/kg i.p.	CBD: ↓ severity of the clinical signs of EAE; ↓ axonal damage and inflammation; ↓ microglial activation and T-cell recruitment in the spinal cord mice	(Kozela et al. 2011)
**Arthritis and osteoarthritis (OA)**				
29	Spontaneous canine OA	Placebo; 20 mg/day (0.5 mg/kg) naked CBD; 50 mg/day (1.2 mg/kg) naked CBD; or 20 mg/day liposomal CBD	CBD: ↓ pain; ↑ mobility	(Verrico et al. 2020)
30	Canine OA	1. Single-dose of CBD enriched (2 or 8 mg/kg) oil<br>2. CBD oil (2 mg/kg) or placebo oil every 12 h for 4 weeks with a 2-week washout period	CBD: ↓ pain; ↑ activity; ↑ alkaline phosphatase in serum	(Gamble et al. 2018)
31	3 mg MIA-induced OA Rat model	Electrophysiological experiments: i.a. infusion of CBD (100, 200, or 300 mg in 100 mL) or vehicle (100 mL)<br>Behavioral experiments: vehicle (50 mL) or CBD (100-300 mg/50 mL) i.a.<br>Inflammation measures: 50-mL bolus of CBD (300 mg) or vehicle topically over the exposed knee joint	Peripheral CBD: ↓ joint afferent firing rate (dose dependently); ↑ withdrawal threshold and weight bearing<br>Local CBD: ↓ transient joint inflammation<br>Prophylactic CBD: prevention of joint pain development; neuroprotective effect<br>CB receptor antagonists AM281 (CB1) and AM630 (CB2): no effect on CBD-induced analgesia<br>TRPV1 antagonist SB-366791: inhibited analgesic effect of CBD	(Philpott, O'Brien, and McDougall 2017)

32	Rat complete Freund's adjuvant-induced monoarthritic knee joint model	CBD transdermal gels (0.6, 3.1, 6.2 or 62.3 mg/day) applied for 4 consecutive days after arthritis induction	CBD 6.2 and 62 mg/kg: ↓ joint swelling, limb posture scores as a rating of spontaneous pain, immune cell infiltration, synovial membrane thickening (dose-dependently); restored paw withdrawal latency; ↓ pro-inflammatory biomarkers in spinal cord (CGRP, OX42) and dorsal root ganglia (TNFα)	(Hammell et al. 2016)
33	Murine collagen-induced arthritis	i.p. daily administration of 20 mg/kg; 10 mg/kg; 5 mg/kg; and 2.5 mg/kg (until day 10 of arthritis)	CBD *in vivo*: ↓ arthritis progression; similar effectiveness when administrated orally and i.p.; ↓ of LPS-induced rise in serum TNFα<br><br>CBD *ex vivo*: ↓ collagen II-specific proliferation (draining lymph node cells from CBD-treated mice); ↓ IFN-γ and TNFα production by knee synovial cells<br><br>CBC *in vitro*: ↓ lymphocyte proliferation (dose-dependently); blockade of the Zymosan-triggered reactive oxygen species in neutrophils	(Malfait et al. 2000)
34	Rheumatoid arthritis synovial fibroblasts *in vitro* culture	CBD (0; 0.5; 1; 5; 10; 20 µM)<br><br>Antagonists tested: A967079 (TRPA1 antagonist); capsazepine (TRPV1 antagonist); cyclosporin A (inhibitor of the mitochondrial permeability transition pore); DIDS (voltage-dependent anion-selective channel inhibitor); Decynium-22 (inhibitor for all organic cation transporter isoforms)	CBD: ↓ cell viability and proliferation; ↓ IL-6, IL-8, MMP-3 production; ↑ intracellular calcium and uptake of the cationic viability dye PoPo3<br><br>A967079 (but not capsazepine): ↓ effects of CBD on calcium and PoPo3 uptake<br><br>Cyclosporin A: blockade of the CBD's effect on cell viability and IL-8 production	(Lowin et al. 2020)

35	Human articular chondrocytes *in vitro* culture	CBD (3-30 or 10-100 µM); AM251 and AM630 (CB1 and CB2 receptors antagonists respectively, 2-16 µM) CdCl2 (Ca2+ channel blocker); Nifedipine (blocker of voltage-gated L-type Ca2+ channels)	CBD: ↓cell viability after (concentrations greater than 4 µM); ↑ caspase 3/7 activity; ↑ in the early apoptotic cell population;↑ elevated $Ca^{2+}{}_i$; depolarization of the cell membrane potential; ↑ Erk1/2 phosphorylation<br><br>AM251: inhibition of the Ca^{2+}; reduction of the toxic effects of CBD	(Winklmayr et al. 2019)
**Other pain models**				
36	Rat model of incision pain	a. Intraperitoneal CBD (0.3-30 mg/kg)<br><br>b. CBD (5-40 nmol/0.25 mL) injected into the rACC	CBD (3 and 10 mg/kg) i.p.: ↓ mechanical allodynia<br><br>CBD (1 and 3 mg/kg) i.p.: reversal of conditioned place preference, produced by peripheral nerve block<br><br>CBD (10-40 nmol/0.25 mL) into the rACC: ↓ mechanical allodynia (dose-dependently)<br><br>CBD (5 nmol/0.25 mL): no change in allodynia; ↓ peripheral nerve block-induced conditioned place preference<br><br>CBD i.p. or into the rACC at doses that did not change the incision pain: conditioned place preference induction	(Genaro et al. 2017)
37	NGF-induced mechanical sensitization in female rats (a rat model of myofascial pain)	Intramuscular (10 µl) injection of: CBD (1 or 5 mg/ml), CBN (1 mg/ml), CBC (1 mg/ml) and CBD/CBN combination (1:1 or 5:1 mg/ml)	CBD or CBN: ↓ NGF-induced mechanical sensitization<br><br>CBD/CBN combinations: ↓ mechanical sensitization (longer-lasting than either compound alone)<br><br>CBD, CBN, CBD/CBN combination: ↑ mechanical threshold of masseter muscle mechanoreceptors (however, CBD/CBN at a higher ratio reduced the duration of this effect)	(Wong and Cairns 2019)

			CBC: no effect on behavioral mechanical withdrawal threshold	
38	Animal models with a critical role of NMDAR overactivity: opioid analgesia attenuation, NMDA-induced convulsive syndrome and ischemic stroke	Intracerebroventricular injection of CBD (10 nmol); morphine (6 nmol); σ1R agonist PPCC (3 nmol); σ1R antagonist BD1063 and progesterone (3 nmol); WAY100635 (5 nmol)	In vivo CBD or BD1063: ↑ morphine-evoked supraspinal antinociception; ↓ NMDA-induced convulsive syndrome; ↓ the infarct size caused by permanent unilateral middle cerebral artery occlusion<br>σ1R knock-out mice and PRE084 and PPCC in WT mice: reduction of CBD's effects	(Rodríguez-Muñoz et al. 2018)
39	3 mouse pain models:<br>a. acetic acid-stimulated stretching<br>b. acetic acid-decreased operant responding for palatable food<br>c. hot plate thermal nociception	a: morphine (0.32–10 mg/kg); CBD (10–40 mg/kg) or its combination<br>b: morphine (0.1–0.32 mg/kg); CBD (5.0–40.0 mg/kg) or its combination<br>c: morphine (1.0–32 mg/kg); CBD (3.2–32 mg/kg) or its combination	Morphine alone: ↓ nociception in all three models<br>CBD alone: ↓ nociception in the acetic acid-stimulated stretching assay only<br>Combinations of CBD and morphine: synergistic effects in reversing acetic acid-stimulated stretching model; subadditive effects in the hot plate thermal nociceptive assay and the acetic acid-decreased operant responding for palatable food assay	(Neelakantan et al. 2015)
40	6-hydroxydopamine-induced Parkinson Disease mouse model	CBD: 10, 30 and 100 mg/kg i.p.<br>Morphine: 10mg/kg i.p.<br>celecoxib (non-steroidal anti-inflammatory): 20mg/kg i.p.	CBD (acute and chronic): ↓ hyperalgesia and allodynia<br>Ineffective doses URB597 or capsazepine: enhancement of the CBD-evoked antinociception	(Crivelaro do Nascimento et al. 2020)

		URB597 (FAAH inhibitor): 0.5mg/kg i.p.<br>AM251 (CB1 antagonist): 1mg/kg i.p.<br>Capsazepine (TRPV1 antagonist): 5mg/kg i.p.<br>SCH336 (CB2 inverse agonist): 2mg/kg i.p.	AM251 or SCH336: prevention of the antinociceptive effect of CBD	
41	Corneal hyperalgesia induced by chemical cauterization of the corneal epithelium in wild-type and CB2R knockout mice	CBD; Δ8-THC; HU-308 dissolved in soybean oil (0.2-5%), topically administered (5 µl) to cauterized corneas 30, 60, and 120 min post cauterization<br>Antagonists tested: AM251 (2 mg/kg i.p.), WAY100635 (1 mg/kg i.p.)	CBD, Δ8-THC, HU-308 in WT mice: ↓ pain score and neutrophil infiltration<br>AM251: blockade of Δ8-THC effect (WT mice)<br>WAY 100635: blockade of CBD effect (WT mice)<br>CBD, Δ8-THC in CB2R knockout mice: ↓ pain score and neutrophil infiltration<br>HU-308 in CB2R knockout mice: no effect on pain score and neutrophil infiltration	(Thapa et al. 2018)
42	Formalin-evoked nociceptive behaviour (rat model of persistent pain)	i.p. preadministration of Δ9-THC (1 or 2.5 mg/kg); CBD (5 mg/kg); morphine (2 mg/kg), Δ9-THC + morphine; Δ9-THC + CBD	Δ9-THC alone: ↓ nociceptive behaviour; ↑ corticosterone response; ↑ the 4-hydroxy-3-methoxyphenylglycol : noradrenaline ratio in the hypothalamus<br>CBD alone: no effect<br>Morphine alone: ↓ nociceptive behaviour<br>Δ9-THC + morphine: ↓ nociceptive behaviour to a greater extent than either drug alone (second phase only); ↑ levels of thalamic 5-hydroxytryptamine; ↓ locomotor activity<br>Δ9-THC + CBD: no modulation of the Δ9-THC effects	(Finn et al. 2004)

Clinical studies				
43	7 patients with kidney transplant-related pain	Increased doses from 50 to 150 mg CBD twice a day for 3 weeks	After 3 weeks of CBD treatment: well-tolerated, mild adverse effects, variable plasma levels of tacrolimus	(Cuñetti et al. 2018)
44	72 children and 60 adults with treatment-resistant epilepsy	CBD 5 mg/kg/day titrated it up to a maximum dosage of 50 mg/kg/day or placebo	CBD: ↓ of the score of Chalfont Seizure Severity Scale (from 80.7 at baseline to 39.2 at 12weeks); ↓ of seizure frequency from a mean of 144.4 to 52.2 at 12 weeks)<br>Similar effects in pediatric and adult groups.	(Szaflarski et al. 2018)
45	225 patients with Lennox–Gastaut syndrome	CBD 10 or 20 mg/kg per day or placebo	CBD: ↓ in seizure frequency (41.9% in the 20 mg/kg CBD group, 37.2% in the 10 mg/kg CBD group, and 17.2% in the placebo group)<br>Adverse events: somnolence, decreased appetite, diarrhea (more frequently in the higher-dose group)	(Devinsky et al. 2018)
46	366 patients with Lennox-Gastaut syndrome	CBD oral solution (Epidiolex; 100 mg/mL), titrated from 2.5 to 20 mg/kg/day or placebo	CBD: ↓ monthly total seizure frequency (from 48% to 57% across all 12-week periods through week 48); ↑ patient's overall condition (88%).<br>The most common adverse effects were diarrhea (26.8%), somnolence (23.5%), and convulsion (21.3%), liver transaminase elevations (10.1%).	(Thiele et al. 2018)

47	607 patients with Lennox-Gastaut syndrome or Dravet syndrome	CBD (Epidiolex®; 100 mg/mL) in oral solution at 2–10 mg/kg/day, titrated until tolerability limit or a maximum dose of 25–50 mg/kg/day.	CBD: ↓ median monthly major motor seizures by 50% and total seizures by 44%<br>Acceptable safety profile; adverse effects: somnolence (30%) and diarrhea (24%)	(Laux et al. 2019)
48	120 children and young adults with the Dravet syndrome and drug-resistant seizures	CBD 20 mg/kg/day or placebo	CBD: ↓ the median frequency of convulsive seizures per month (from 12.4 to 5.9); ↑ patient's overall condition	(Devinsky et al. 2017)
49	97 patients with chronic pain (at least 3 years) who have been on opioids for at least 1 year	CDB gels containing 15.7 mg CBD, 0.5 mg THC, 0.3 mg cannabidivarin (CBDV), 0.9 mg cannabidiolic acid (CBDA), 0.8 mg cannabichrome (CBC), and >1% botanical terpene blend<br>Almost all participants (91) used two soft gels (~30 mg) daily	After 8 weeks of treatment: elimination of opioid use in 53% of patients; improvement of the quality of life in 94% of patients; significant relationship between CBD and Pittsburgh Sleep Quality Index and Pain Intensity and Interference	(Capano, Weaver, and Burkman 2020)
50	20 chronic pain patients with fibromyalgia	Single vapor inhalation of 4 different cannabis varieties: Bedrocan (22.4 mg Δ9-THC, <1 mg CBD), Bediol (13.4 mg Δ9-THC, 17.8 mg CBD, Bedrolite (18.4 mg CBD, <1 mg Δ9-THC), placebo (no CBB or Δ9-THC)	All drugs: significant effect on spontaneous or electrical pain responses (although more subjects receiving Bediol displayed a 30% decrease in pain scores compared to placebo)<br>Cannabis varieties containing THC: ↑ pressure pain threshold	(Van De Donk et al. 2019)
51	20 multiple sclerosis patients (10 with and 10 without neuropathic pain)	Cannabis-based medicine extract (Sativex) in a spray containing THC (27 mg/mL) and CBD (25 mg/mL), with ethanol/propylene glycol (50:50) excipient. Each	After 4 weeks of Sativex treatment in neuropathic pain patients: ↓ rating; ↑ quality of life (effects paralleled by an increase of fronto-central γ-band oscillation and pain-motor integration strength)	(Russo et al. 2016)

		actuation delivers 100 mL of spray, mean number of sprays daily administered was eight.		
52	16 patients with chemotherapy-induced neuropathic pain	Nabiximols, an oral mucosal spray combining Δ9-THC with CBD, minor cannabinoids and terpenoids, ethanol and propylene glycol excipients, and peppermint flavoring	After 4 weeks of Nabiximols treatment: no significant difference in the pain intensity between the treatment and placebo groups<br>Five participants reported a two-point or greater reduction in pain	(Lynch, Cesar-Rittenberg, and Hohmann 2014)
53	234 patients with peripheral neuropathic pain or allodynia associated with diabetes	Δ9-THC/CBD oral mucosal spray for a 38 weeks in addition to current analgesic therapy, each 100 µL actuation of spray delivered 2.7 mg of THC and 2.5 mg of CBD (maximum of eight sprays per 3 h period and 24 actuations every 24 h)	Δ9-THC/CBD group after 38 weeks of treatment: ↓ pain score (from mean 6.9 to 4.2); at least half of all patients reported a 30 % improvement at all time points in sleep quality, neuropathic pain, subject global impression of change and EQ-5D questionnaire	(Hoggart et al. 2015)
54	303 patients with peripheral neuropathic pain and allodynia	Self-administered oral mucosal spray (100 µL) containing 2.7 mg of Δ9-THC and 2.5 mg of CBD (maximum of eight sprays in a 3 h period up to a maximum of 24 sprays per 24 h period)	Δ9-THC/CBD group after 24 weeks of treatment: ↓ pain score; ↑ sleep quality and Subject Global Impression of Change	(Serpell et al. 2014)
55	177 patients with cancer-related pain experiencing inadequate analgesia despite chronic opioid dosing	100 µL actuation of Δ9-THC/CBD spray (2.7 mg of THC and 2.5 mg of CBD); Δ9-THC (2.7 mg of Δ9-THC) or placebo (excipients plus colorants) to the oral mucosa self-administered to patient's optimal dose (maximum of eight actuations in a 3 h period up to a maximum of 48 sprays per 24 h period)	Δ9-THC/CBD group after 2 weeks of treatment: ↓ pain in Numeral Rating Scale; reduction of more than 30% from baseline pain (twice as many patients in comparison to placebo); nausea and vomiting side effects<br>Δ9-THC group after 2 weeks of treatment: similar to placebo	(Johnson et al. 2010)

56	43 patients with cancer-related pain experiencing inadequate analgesia despite chronic opioid dosing	100 µL actuation of Δ9-THC/CBD spray (2.7 mg of THC and 2.5 mg of CBD); Δ9-THC (2.7 mg of Δ9-THC) or placebo (excipients plus colorants) to the oral mucosa self-administered to patient's optimal dose (maximum of eight actuations in a 3 h period up to a maximum of 48 sprays per 24 h period)	Δ9-THC/CBD spray patients: ↓ pain severity, insomnia and fatigue	(Johnson et al. 2013)
57	29 patients with symptomatic peripheral neuropathy	Topical Theramu Relieve CBD compound cream (250 mg of CBD per 3 fl. oz), or placebo emu oil cream, applied topically to the symptomatic areas up to four times per day during 4 weeks	CBD group after 4 weeks of treatment: ↓ intense pain, sharp pain, cold and itchy sensations (compared to the placebo); no adverse events	(Xu et al. 2019)
58	263 patients with advanced cancer and opioid-refractory pain	Nabiximols at a low dose (1–4 sprays/day), medium dose (6–10 sprays/day), or high dose (11–16 sprays/day). Each actuation delivered 100 mL of fluid to the oral mucosa contained 2.7 mg Δ9-THC and 2.5 mg CBD	After 35 days of treatment: proportion of patients reporting analgesia was greater for nabiximols than placebo overall, and specifically in the low-dose and medium-dose groups (secondary continuous responder analysis of average); questionnaires showed no significant group differences; adverse effects in a high-dose group only	(Portenoy et al. 2012)

Abraham, Antony D., Edward J.Y. Leung, Brenden A. Wong, Zeena M.G. Rivera, Lauren C. Kruse, Jeremy J. Clark, and Benjamin B. Land. 2020. "Orally Consumed Cannabinoids Provide Long-Lasting Relief of Allodynia in a Mouse Model of Chronic Neuropathic Pain." *Neuropsychopharmacology* 45 (7): 1105–14. https://doi.org/10.1038/s41386-019-0585-3.

Britch, Stevie C., Alan G. Goodman, Jenny L. Wiley, Abby M. Pondelick, and Rebecca M. Craft. 2020. "Antinociceptive and Immune Effects of Delta-9-Tetrahydrocannabinol or Cannabidiol in Male Versus Female Rats with Persistent Inflammatory Pain." *Journal of Pharmacology and Experimental Therapeutics* 373 (3): 416–28. https://doi.org/10.1124/jpet.119.263319.

Britch, Stevie C., Jenny L. Wiley, Zhihao Yu, Brian H. Clowers, and Rebecca M. Craft. 2017. "Cannabidiol-Δ9-Tetrahydrocannabinol Interactions on Acute Pain

and Locomotor Activity." *Drug and Alcohol Dependence* 175 (June): 187–97. https://doi.org/10.1016/j.drugalcdep.2017.01.046.

Capano, Alex, Richard Weaver, and Elisa Burkman. 2020. "Evaluation of the Effects of CBD Hemp Extract on Opioid Use and Quality of Life Indicators in Chronic Pain Patients: A Prospective Cohort Study." *Postgraduate Medicine* 132 (1): 56–61. https://doi.org/10.1080/00325481.2019.1685298.

Casey, Sherelle L., Nicholas Atwal, and Christopher W. Vaughan. 2017. "Cannabis Constituent Synergy in a Mouse Neuropathic Pain Model." *Pain* 158 (12): 2452–60. https://doi.org/10.1097/j.pain.0000000000001051.

Costa, Barbara, Anna Elisa Trovato, Francesca Comelli, Gabriella Giagnoni, and Mariapia Colleoni. 2007. "The Non-Psychoactive Cannabis Constituent Cannabidiol Is an Orally Effective Therapeutic Agent in Rat Chronic Inflammatory and Neuropathic Pain." *European Journal of Pharmacology* 556 (1–3): 75–83. https://doi.org/10.1016/j.ejphar.2006.11.006.

Crivelaro do Nascimento, Glauce, Daniele Pereira Ferrari, Francisco Silveira Guimaraes, Elaine Aparecida Del Bel, Mariza Bortolanza, and Nilson Carlos Ferreira- Junior. 2020. "Cannabidiol Increases the Nociceptive Threshold in a Preclinical Model of Parkinson's Disease." *Neuropharmacology* 163 (February). https://doi.org/10.1016/j.neuropharm.2019.107808.

Cuñetti, L., L. Manzo, R. Peyraube, J. Arnaiz, L. Curi, and S. Orihuela. 2018. "Chronic Pain Treatment With Cannabidiol in Kidney Transplant Patients in Uruguay." *Transplantation Proceedings* 50 (2): 461–64. https://doi.org/10.1016/j.transproceed.2017.12.042.

Devinsky, Orrin, J. Helen Cross, Linda Laux, Eric Marsh, Ian Miller, Rima Nabbout, Ingrid E. Scheffer, Elizabeth A. Thiele, and Stephen Wright. 2017. "Trial of Cannabidiol for Drug-Resistant Seizures in the Dravet Syndrome." *New England Journal of Medicine*. https://doi.org/10.1056/NEJMoa1611618.

Devinsky, Orrin, Anup D. Patel, J. Helen Cross, Vicente Villanueva, Elaine C. Wirrell, Michael Privitera, Sam M. Greenwood, et al. 2018. "Effect of Cannabidiol on Drop Seizures in the Lennox–Gastaut Syndrome." *New England Journal of Medicine*. https://doi.org/10.1056/NEJMoa1714631.

Donk, Tine Van De, Marieke Niesters, Mikael A. Kowal, Erik Olofsen, Albert Dahan, and Monique Van Velzen. 2019. "An Experimental Randomized Study on the Analgesic Effects of Pharmaceutical-Grade Cannabis in Chronic Pain Patients with Fibromyalgia." *Pain* 160 (4): 860–69. https://doi.org/10.1097/j.pain.0000000000001464.

Finn, D. P., S. R.G. Beckett, C. H. Roe, A. Madjd, K. C.F. Fone, D. A. Kendall, C. A. Marsden, and V. Chapman. 2004. "Effects of Coadministration of Cannabinoids and Morphine on Nociceptive Behaviour, Brain Monoamines and HPA Axis Activity in a Rat Model of Persistent Pain." *European Journal of Neuroscience*. https://doi.org/10.1111/j.0953-816X.2004.03177.x.

Gamble, Lauri-Jo, Jordyn M. Boesch, Christopher W. Frye, Wayne S. Schwark, Sabine Mann, Lisa Wolfe, Holly Brown, Erin S. Berthelsen, and Joseph J. Wakshlag. 2018. "Pharmacokinetics, Safety, and Clinical Efficacy of Cannabidiol Treatment in Osteoarthritic Dogs." *Frontiers in Veterinary Science* 5 (July). https://doi.org/10.3389/fvets.2018.00165.

Genaro, Karina, Débora Fabris, Ana L.F. Arantes, Antônio W. Zuardi, José A.S. Crippa, and Wiliam A. Prado. 2017. "Cannabidiol Is a Potential Therapeutic for

the Affective-Motivational Dimension of Incision Pain in Rats." *Frontiers in Pharmacology* 8 (JUN). https://doi.org/10.3389/fphar.2017.00391.

Gonçalves, Thais Cristina Teixeira, Anna Karla Londe, Rafael Isaac Pires Albano, Artur Teixeira De Araújo Júnior, Mariana De Aguiar Azeredo, Audrey Francisco Biagioni, Thiago Henrique Ferreira Vasconcellos, et al. 2014. "Cannabidiol and Endogenous Opioid Peptide-Mediated Mechanisms Modulate Antinociception Induced by Transcutaneous Electrostimulation of the Peripheral Nervous System." *Journal of the Neurological Sciences* 347 (1–2): 82–89. https://doi.org/10.1016/j.jns.2014.09.024.

Greene, Nicholas Z., Jenny L. Wiley, Zhihao Yu, Brian H. Clowers, and Rebecca M. Craft. 2018. "Cannabidiol Modulation of Antinociceptive Tolerance to Δ9-Tetrahydrocannabinol." *Psychopharmacology* 235 (11): 3289–3302. https://doi.org/10.1007/s00213-018-5036-z.

Gregorio, Danilo De, Ryan J. McLaughlin, Luca Posa, Rafael Ochoa-Sanchez, Justine Enns, Martha Lopez-Canul, Matthew Aboud, Sabatino Maione, Stefano Comai, and Gabriella Gobbi. 2019. "Cannabidiol Modulates Serotonergic Transmission and Reverses Both Allodynia and Anxiety-like Behavior in a Model of Neuropathic Pain." *Pain* 160 (1): 136–50. https://doi.org/10.1097/j.pain.0000000000001386.

Hammell, D. C., L. P. Zhang, F. Ma, S. M. Abshire, S. L. McIlwrath, A. L. Stinchcomb, and K. N. Westlund. 2016. "Transdermal Cannabidiol Reduces Inflammation and Pain-Related Behaviours in a Rat Model of Arthritis." *European Journal of Pain (United Kingdom)* 20 (6): 936–48. https://doi.org/10.1002/ejp.818.

Harris, Hannah M., Kenneth J. Sufka, Waseem Gul, and Mahmoud A. Elsohly. 2016. "Effects of Delta-9-Tetrahydrocannabinol and Cannabidiol on Cisplatin-Induced Neuropathy in Mice." *Planta Medica* 82 (13): 1169–72. https://doi.org/10.1055/s-0042-106303.

Hoggart, B., S. Ratcliffe, E. Ehler, K. H. Simpson, J. Hovorka, J. Lejčko, L. Taylor, H. Lauder, and M. Serpell. 2015. "A Multicentre, Open-Label, Follow-on Study to Assess the Long-Term Maintenance of Effect, Tolerance and Safety of THC/CBD Oromucosal Spray in the Management of Neuropathic Pain." *Journal of Neurology* 262 (1): 27–40. https://doi.org/10.1007/s00415-014-7502-9.

Jesus, Carlos Henrique Alves, Daiany Darlly Bello Redivo, Aléxia Thamara Gasparin, Bruna Bittencourt Sotomaior, Milene Cristina de Carvalho, Karina Genaro, Antonio Waldo Zuardi, et al. 2019. "Cannabidiol Attenuates Mechanical Allodynia in Streptozotocin-Induced Diabetic Rats via Serotonergic System Activation through 5-HT1A Receptors." *Brain Research* 1715 (July): 156–64. https://doi.org/10.1016/j.brainres.2019.03.014.

Johnson, Jeremy R., Mary Burnell-Nugent, Dominique Lossignol, Elena Doina Ganae-Motan, Richard Potts, and Marie T. Fallon. 2010. "Multicenter, Double-Blind, Randomized, Placebo-Controlled, Parallel-Group Study of the Efficacy, Safety, and Tolerability of THC:CBD Extract and THC Extract in Patients with Intractable Cancer-Related Pain." *Journal of Pain and Symptom Management* 39 (2): 167–79. https://doi.org/10.1016/j.jpainsymman.2009.06.008.

Johnson, Jeremy R., Dominique Lossignol, Mary Burnell-Nugent, and Marie T. Fallon. 2013. "An Open-Label Extension Study to Investigate the Long-Term Safety and Tolerability of THC/CBD Oromucosal Spray and Oromucosal THC Spray in Patients with Terminal Cancer-Related Pain Refractory to Strong Opioid Analgesics." *Journal of Pain and Symptom Management* 46 (2): 207–18. https://doi.org/10.1016/j.jpainsymman.2012.07.014.

Karmaus, Peer W.F., James G. Wagner, Jack R. Harkema, Norbert E. Kaminski, and Barbara L.F. Kaplan. 2013. "Cannabidiol (CBD) Enhances Lipopolysaccharide (LPS)-Induced Pulmonary Inflammation in C57BL/6 Mice." *Journal of Immunotoxicology* 10 (3): 321–28. https://doi.org/10.3109/1547691X.2012.741628.

King, Kirsten M., Alyssa M. Myers, Ariele J. Soroka-Monzo, Ronald F. Tuma, Ronald J. Tallarida, Ellen A. Walker, and Sara Jane Ward. 2017. "Single and Combined Effects of Δ9-Tetrahydrocannabinol and Cannabidiol in a Mouse Model of Chemotherapy-Induced Neuropathic Pain." *British Journal of Pharmacology* 174 (17): 2832–41. https://doi.org/10.1111/bph.13887.

Kozela, Ewa, Nirit Lev, Nathali Kaushansky, Raya Eilam, Neta Rimmerman, Rivka Levy, Avraham Ben-Nun, Ana Juknat, and Zvi Vogel. 2011. "Cannabidiol Inhibits Pathogenic T Cells, Decreases Spinal Microglial Activation and Ameliorates Multiple Sclerosis-like Disease in C57BL/6 Mice." *British Journal of Pharmacology*. https://doi.org/10.1111/j.1476-5381.2011.01379.x.

Laux, Linda C., E. Martina Bebin, Daniel Checketts, Michael Chez, Robert Flamini, Eric D. Marsh, Ian Miller, et al. 2019. "Long-Term Safety and Efficacy of Cannabidiol in Children and Adults with Treatmentresistant Lennox-Gastaut Syndrome or Dravet Syndrome: Expanded Access Program Results." *Epilepsy Research*. https://doi.org/10.1016/j.eplepsyres.2019.03.015.

Lehmann, Christian, Nicholas B. Fisher, Barna Tugwell, Anna Szczesniak, Mel Kelly, and Juan Zhou. 2016. "Experimental Cannabidiol Treatment Reduces Early Pancreatic Inflammation in Type 1 Diabetes." *Clinical Hemorheology and Microcirculation* 64 (4): 655–62. https://doi.org/10.3233/CH-168021.

Li, Hongbo, Weimin Kong, Christina R. Chambers, Daohai Yu, Doina Ganea, Ronald F. Tuma, and Sara Jane Ward. 2018. "The Non-Psychoactive Phytocannabinoid Cannabidiol (CBD) Attenuates pro-Inflammatory Mediators, T Cell Infiltration, and Thermal Sensitivity Following Spinal Cord Injury in Mice." *Cellular Immunology* 329 (July): 1–9. https://doi.org/10.1016/j.cellimm.2018.02.016.

Linher-Melville, Katja, Yong Fang Zhu, Jesse Sidhu, Natalka Parzei, Ayesha Shahid, Gireesh Seesankar, Danny Ma, et al. 2020. "Evaluation of the Preclinical Analgesic Efficacy of Naturally Derived, Orally Administered Oil Forms of Δ9-Tetrahydrocannabinol (THC), Cannabidiol (CBD), and Their 1:1 Combination." *PLoS ONE* 15 (6). https://doi.org/10.1371/journal.pone.0234176.

Lowin, Torsten, Ren Tingting, Julia Zurmahr, Tim Classen, Matthias Schneider, and Georg Pongratz. 2020. "Cannabidiol (CBD): A Killer for Inflammatory Rheumatoid Arthritis Synovial Fibroblasts." *Cell Death and Disease* 11 (714). https://doi.org/10.1038/s41419-020-02892-1.

Lynch, Mary E., Paula Cesar-Rittenberg, and Andrea G. Hohmann. 2014. "A Double-Blind, Placebo-Controlled, Crossover Pilot Trial with Extension Using an Oral Mucosal Cannabinoid Extract for Treatment of Chemotherapy-Induced Neuropathic Pain." *Journal of Pain and Symptom Management* 47 (1): 166–73. https://doi.org/10.1016/j.jpainsymman.2013.02.018.

Maione, Sabatino, Fabiana Piscitelli, Luisa Gatta, Daniela Vita, Luciano De Petrocellis, Enza Palazzo, Vito De Novellis, and Vincenzo Di Marzo. 2011. "Non-Psychoactive Cannabinoids Modulate the Descending Pathway of Antinociception in Anaesthetized Rats through Several Mechanisms of Action." *British Journal of Pharmacology* 162 (3): 584–96. https://doi.org/10.1111/j.1476-5381.2010.01063.x.

Malfait, A. M., R. Gallily, P. F. Sumariwalla, A. S. Malik, E. Andreakos, R. Mechoulam, and M. Feldmann. 2000. “The Nonpsychoactive Cannabis Constituent Cannabidiol Is an Oral Anti-Arthritic Therapeutic in Murine Collagen-Induced Arthritis.” *Proceedings of the National Academy of Sciences of the United States of America*. https://doi.org/10.1073/pnas.160105897.

Moreno-Martet, Miguel, Ana Feliú, Francisco Espejo-Porras, Miriam Mecha, Francisco J. Carrillo-Salinas, Javier Fernández-Ruiz, Carmen Guaza, and Eva De Lago. 2015. “The Disease-Modifying Effects of a Sativex-like Combination of Phytocannabinoids in Mice with Experimental Autoimmune Encephalomyelitis Are Preferentially Due to Δ9-Tetrahydrocannabinol Acting through CB1 Receptors.” *Multiple Sclerosis and Related Disorders* 4 (6): 505–11. https://doi.org/10.1016/j.msard.2015.08.001.

Neelakantan, Harshini, Ronald J. Tallarida, Zachary W. Reichenbach, Ronald F. Tuma, Sara J. Ward, and Ellen A. Walker. 2015. “Distinct Interactions of Cannabidiol and Morphine in Three Nociceptive Behavioral Models in Mice.” *Behavioural Pharmacology* 26 (3): 304–14. https://doi.org/10.1097/FBP.0000000000000119.

Pan, Hao, Partha Mukhopadhyay, Mohanraj Rajesh, Vivek Patel, Bani Mukhopadhyay, Bin Gao, György Haskó, and Pál Pacher. 2009. “Cannabidiol Attenuates Cisplatin-Lnduced Nephrotoxicity by Decreasing Oxidative/Nitrosative Stress, Inflammation, and Cell Death.” *Journal of Pharmacology and Experimental Therapeutics* 328 (3): 708–14. https://doi.org/10.1124/jpet.108.147181.

Philpott, Holly T., Melissa O’Brien, and Jason J. McDougall. 2017. “Attenuation of Early Phase Inflammation by Cannabidiol Prevents Pain and Nerve Damage in Rat Osteoarthritis.” *Pain* 158 (12): 2442–51. https://doi.org/10.1097/j.pain.0000000000001052.

Portenoy, Russell K., Elena Doina Ganae-Motan, Silvia Allende, Ronald Yanagihara, Lauren Shaiova, Sharon Weinstein, Robert McQuade, Stephen Wright, and Marie T. Fallon. 2012. “Nabiximols for Opioid-Treated Cancer Patients with Poorly-Controlled Chronic Pain: A Randomized, Placebo-Controlled, Graded-Dose Trial.” *Journal of Pain* 13 (5): 438–49. https://doi.org/10.1016/j.jpain.2012.01.003.

Rock, Erin M., Cheryl L. Limebeer, and Linda A. Parker. 2018. “Effect of Cannabidiolic Acid and Δ9-Tetrahydrocannabinol on Carrageenan-Induced Hyperalgesia and Edema in a Rodent Model of Inflammatory Pain.” *Psychopharmacology* 235 (11): 3259–71. https://doi.org/10.1007/s00213-018-5034-1.

Rodríguez-Muñoz, María, Yara Onetti, Elsa Cortés-Montero, Javier Garzón, and Pilar Sánchez-Blázquez. 2018. “Cannabidiol Enhances Morphine Antinociception, Diminishes NMDA-Mediated Seizures and Reduces Stroke Damage via the Sigma 1 Receptor 11 Medical and Health Sciences 1109 Neurosciences 11 Medical and Health Sciences 1115 Pharmacology and Pharmaceutical Sciences.” *Molecular Brain* 11 (1). https://doi.org/10.1186/s13041-018-0395-2.

Russo, Margherita, Antonino Naro, Antonino Leo, Edoardo Sessa, Giangaetano D’Aleo, Placido Bramanti, and Rocco Salvatore Calabrò. 2016. “Evaluating Sativex® in Neuropathic Pain Management: A Clinical and Neurophysiological Assessment in Multiple Sclerosis.” *Pain Medicine (United States)* 17 (6): 1145–54. https://doi.org/10.1093/pm/pnv080.

Serpell, Mick, S. Ratcliffe, J. Hovorka, M. Schofield, L. Taylor, H. Lauder, and E. Ehler. 2014. “A Double-Blind, Randomized, Placebo-Controlled, Parallel Group Study of THC/CBD Spray in Peripheral Neuropathic Pain Treatment.” *European Journal of Pain (United Kingdom)* 18 (7): 999–1012. https://doi.org/10.1002/j.1532-2149.2013.00445.x.

Szaflarski, Jerzy P., Elizabeth Martina Bebin, Gary Cutter, Jennifer DeWolfe, Leon S. Dure, Tyler E. Gaston, Pongkiat Kankirawatana, et al. 2018. “Cannabidiol Improves Frequency and Severity of Seizures and Reduces Adverse Events in an Open-Label Add-on Prospective Study.” *Epilepsy and Behavior*. https://doi.org/10.1016/j.yebeh.2018.07.020.

Thapa, Dinesh, Elizabeth A. Cairns, Anna Maria Szczesniak, James T. Toguri, Meggie D. Caldwell, and Melanie E.M. Kelly. 2018. “The Cannabinoids Δ8THC, CBD, and HU-308 Act via Distinct Receptors to Reduce Corneal Pain and Inflammation.” *Cannabis and Cannabinoid Research* 3 (1): 11–20. https://doi.org/10.1089/can.2017.0041.

Thiele, Elizabeth A., Eric D. Marsh, Jacqueline A. French, Maria B. Mazurkiewicz, Selim R. Benbadis, Charuta Joshi, Paul D. Lyons, et al. 2018. “Cannabidiol in Patients with Seizures Associated with Lennox-Gastaut Syndrome (GWPCARE4): A Randomised, Double-Blind, Placebo-Controlled Phase 3 Trial.” *The Lancet*. https://doi.org/10.1016/S0140-6736(18)30136-3.

Toth, Cory C., Nicole M. Jedrzejewski, Connie L. Ellis, and William H. Frey. 2010. “Cannabinoid-Mediated Modulation of Neuropathic Pain and Microglial Accumulation in a Model of Murine Type I Diabetic Peripheral Neuropathic Pain.” *Molecular Pain* 6 (March). https://doi.org/10.1186/1744-8069-6-16.

Verrico, Chris D, Shonda Wesson, Vanaja Konduri, Colby J Hofferek, Jonathan Vazquez-Perez, Emek Blair, Kenneth Dunner, Pedram Salimpour, William K Decker, and Matthew M Halpert. 2020. “A Randomized, Double-Blind, Placebo-Controlled Study of Daily Cannabidiol for the Treatment of Canine Osteoarthritis Pain.” https://doi.org/10.1097/j.pain.0000000000001896.

Vigil, Jacob M., Marena A. Montera, Nathan S. Pentkowski, Jegason P. Diviant, Joaquin Orozco, Anthony L. Ortiz, Lawrence J. Rael, and Karin N. Westlund. 2020. “The Therapeutic Effectiveness of Full Spectrum Hemp Oil Using a Chronic Neuropathic Pain Model.” *Life* 10 (5). https://doi.org/10.3390/life10050069.

Ward, Sara Jane, Sean D. McAllister, Rumi Kawamura, Ryuchi Murase, Harshini Neelakantan, and Ellen A. Walker. 2014. “Cannabidiol Inhibits Paclitaxel-Induced Neuropathic Pain through 5-HT 1A Receptors without Diminishing Nervous System Function or Chemotherapy Efficacy.” *British Journal of Pharmacology* 171 (3): 636–45. https://doi.org/10.1111/bph.12439.

Ward, Sara Jane, Michael David Ramirez, Harshini Neelakantan, and Ellen Ann Walker. 2011. “Cannabidiol Prevents the Development of Cold and Mechanical Allodynia in Paclitaxel-Treated Female C57bl6 Mice.” *Anesthesia and Analgesia* 113 (4): 947–50. https://doi.org/10.1213/ANE.0b013e3182283486.

Winklmayr, Martina, Martin Gaisberger, Michael Kittl, Julia Fuchs, Markus Ritter, and Martin Jakab. 2019. “Dose-Dependent Cannabidiol-Induced Elevation of Intracellular Calcium and Apoptosis in Human Articular Chondrocytes.” *Journal of Orthopaedic Research*, August. https://doi.org/10.1002/jor.24430.

Wong, Hayes, and Brian E. Cairns. 2019. “Cannabidiol, Cannabinol and Their Combinations Act as Peripheral Analgesics in a Rat Model of Myofascial Pain.” *Archives of Oral Biology* 104 (August): 33–39. https://doi.org/10.1016/j.archoralbio.2019.05.028.

Xiong, Wei, Tanxing Cui, Kejun Cheng, Fei Yang, Shao Rui Chen, Dan Willenbring, Yun Guan, et al. 2012. “Cannabinoids Suppress Inflammatory and Neuropathic Pain by Targeting A3 Glycine Receptors.” *Journal of Experimental Medicine* 209 (6): 1121–34. https://doi.org/10.1084/jem.20120242.

Xu, Dixon H., Benjamin D. Cullen, Meng Tang, and Yujiang Fang. 2019. “The Effectiveness of Topical Cannabidiol Oil in Symptomatic Relief of Peripheral Neuropathy of the Lower Extremities.” *Current Pharmaceutical Biotechnology* 21 (5): 390–402. https://doi.org/10.2174/1389201020666191202111534.
